# Imposing Dirichlet boundary conditions directly for FFT-based computational micromechanics

**DOI:** 10.1007/s00466-024-02469-1

**Published:** 2024-06-07

**Authors:** Lennart Risthaus, Matti Schneider

**Affiliations:** 1 Institute of Engineering Mathematics, University of Duisburg-Essen, Essen, Germany; 2 Fraunhofer Institute for Industrial Mathematics ITWM, Kaiserslautern, Germany

**Keywords:** FFT-based computational micromechanics, Dirichlet boundary conditions, Discrete sine transform, Discrete cosine transform, Computational homogenization

## Abstract

We discuss how Dirichlet boundary conditions can be directly imposed for the Moulinec–Suquet discretization on the boundary of rectangular domains in iterative schemes based on the fast Fourier transform (FFT) and computational homogenization problems in mechanics. Classically, computational homogenization methods based on the fast Fourier transform work with periodic boundary conditions. There are applications, however, when Dirichlet (or Neumann) boundary conditions are required. For thermal homogenization problems, it is straightforward to impose such boundary conditions by using discrete sine (and cosine) transforms instead of the FFT. This approach, however, is not readily extended to mechanical problems due to the appearance of mixed derivatives in the Lamé operator of elasticity. Thus, Dirichlet boundary conditions are typically imposed either by using Lagrange multipliers or a “buffer zone” with a high stiffness. Both strategies lead to formulations which do not share the computational advantages of the original FFT-based schemes. The work at hand introduces a technique for imposing Dirichlet boundary conditions directly without the need for indefinite systems. We use a formulation on the deformation gradient—also at small strains—and employ the Green’s operator associated to the vector Laplacian. Then, we develop the Moulinec–Suquet discretization for Dirichlet boundary conditions—requiring carefully selected weights at boundary points—and discuss the seamless integration into existing FFT-based computational homogenization codes based on dedicated discrete sine/cosine transforms. The article culminates with a series of well-chosen numerical examples demonstrating the capabilities of the introduced technology.

## Introduction

### State of the art

Computational homogenization methods are widely-used to determine the material behavior of materials with heterogeneous microstructure [1, 55]. A particularly efficient approach to computational micromechanics is based on the fast Fourier transform (FFT) and was introduced by Moulinec–Suquet in their seminal articles [65, 66]. Their strategy was focused on small-strain nonlinear and inelastic constitutive laws and used a Lippmann–Schwinger formulation [44, 67, 103] for periodic homogenization without inertial effects and body forces. The strategy rested upon a discretization on a regular grid, ensuring compatibility to microstructure images obtained by micro-computed tomography ($\mu$-CT, [11, 18]), was formulated on the strain field in the first place and made heavy use of the fast Fourier transform, leveraging the capabilities of well-implemented FFT software packages.

The apparent computational power of this FFT-based approach to computational micromechanics led to a number of subsequent developments which tried to preserve the efficiency of the original method while fixing a few shortcomings of the original method. Some of the earliest contributions realized that the solution method introduced in the original articles [65, 66] and called *basic scheme* by the authors, requires an iteration count proportional to the material contrast. In particular, for high material contrast, the required iteration count could be massive. As a remedy, several enhanced solution strategies were introduced. Based on an ingenious reformulation of the Lippmann–Schwinger equation, Eyre–Milton [21, 92] and Michel–Moulinec–Suquet [56, 57] introduced solution schemes operating on the polarization stress instead of the strain. For elastic materials with finite contrast, these methods were found to converge with the square root of the material contrast for proper choice of numerical parameters, offering a significant upgrade to the original basic scheme. Later on, the polarization methods were revisited, unified and thoroughly analyzed [59, 60, 64], in particular for the case of nonlinear materials [80, 86]. Unfortunately, their range of applicability is limited, yet these methods will shine more often than not if applicable [19, 100].

A second line of solver extensions made use of Newton’s method [46] in this framework, which received a significant boost [12, 28, 38] when combined with Krylov solvers [7, 104]. More recent contributions analyzed the impact of dedicated Quasi-Newton methods [9, 96, 97], which remove the user both from the necessity to implement the material tangent and the arduous task of selecting the solver specifications appropriately.

The third line of solver extensions is based on a re-interpretation of Moulinec–Suquet’s basic scheme as a gradient-descent method [38, 78]. This unexpected connection to the world of optimization permitted to import battle-tested optimization methods into FFT-based computational micromechanics like accelerated gradient [20, 74] as well as conjugate gradient methods [77, 104] or the celebrated Barzilai-Borwein scheme [2, 75].

Usually, the advent of solvers went hand-in-hand with extensions of the applicability of FFT-based solution schemes. For instance, extensions to finite elasticity [29, 46, 54], crystal plasticity [17, 47, 91], damage and fracture mechanics [10, 50, 62] and many others were reported. We refer to the dedicated review articles [48, 79, 88] for a glimpse at the numerous applications of FFT-based schemes.

In a complementary direction, different discretization schemes were investigated by the community. This interest was mainly driven by trying to remove the spurious oscillations visible in the solution fields of the original Moulinec–Suquet discretization [65, 66]. However, F. Willot [99] was probably the first to realize that using certain finite-difference discretizations permitted to apply FFT-based solution schemes to mechanically stable porous microstructures, i.e., to materials with *infinite* material contrast. This observation was later analyzed from a mathematical point of view [78].

There is quite a number of discretization schemes in FFT-based computational micromechanics which fit into the Lippmann–Schwinger framework and operate on a regular grid: Fourier-Galerkin discretizations [6, 58, 93], the discretization with voxel-wise constant strains [7, 8, 76] and various finite-difference [84, 99, 101] and finite-element [45, 49, 85] discretizations, see also Zeman et al. [105]. Again, we refer to the dedicated review articles [48, 79, 88] for more background and a historical discussion.

The use of the fast Fourier transform meant that the natural boundary conditions for the displacement-fluctuation field on the micro-scale are *periodic* boundary conditions. These conditions are a perfect fit for cellular materials [13, 53], but turn out to be advantageous for materials with random microstructure [16, 35, 87], as well. In contrast to FFT-based methods, displacement (Dirichlet) or normal-stress (Neumann) boundary conditions are more frequently used in the finite element (FE) method, and imposing periodic boundary conditions requires a special treatment [89].

There are certain applications, however, where using such non-periodic boundary conditions would be favorable or even required by the application at hand. As an example, Bödecker et al. [3, 4] consider a compression test of a composite plate where Dirichlet boundary conditions are essential to match the experimental setup. Actually, there appears to be a more straightforward solution to the problem. FFT-based computational homogenization methods critically rely upon (a discretized version of) Green’s operator for the underlying physical problem. In small-strain mechanics, it is necessary to solve the mechanical problem

1 $$\begin{aligned} 2\mu ^0 \Delta \varvec{ u}+ (\mu ^0 + \lambda ^0) \nabla \text {div }\varvec{ u}= \varvec{ f}\end{aligned}$$

for the displacement field $\varvec{ u}$, where the body-force field $\varvec{ f}$ and the Lame parameters $\mu ^0$ and $\lambda ^0$ are fixed. If periodic boundary conditions for the field $\varvec{ u}$ are considered, we may use a representation by Fourier series

2 $$\begin{aligned} \varvec{ u}(\varvec{ x})&= \sum _{\varvec{\xi } \in \mathbb {Z}^d} \hat{\varvec{ u}}(\varvec{\xi }) \, e^{i \, \varvec{\xi } \cdot \varvec{x}},\nonumber \\ {\varvec{f}}({\varvec{x}})&= \sum _{\varvec{\xi } \in \mathbb {Z}^d} \hat{\varvec{ f}}(\varvec{\xi }) \, e^{i \, \varvec{\xi } \cdot \varvec{x}}, \quad \varvec{ x}\in Y, \end{aligned}$$

with suitable Fourier coefficients $\hat{\varvec{ u}}(\varvec{\xi })$ and $\hat{\varvec{ f}}(\varvec{\xi })$ and where we consider the cell $Y = [0,2\pi ]^d$ in $d=2,3$ dimensions. Then, the action of the Lamé operator (1) on the field $\varvec{ u}$ leads to the expression

3 $$\begin{aligned}&\mu ^0 \Delta \varvec{ u}+ (\mu ^0 + \lambda ^0) \nabla \text {div }\varvec{ u}(\varvec{ x})\nonumber \\&\quad = -\sum _{\varvec{\xi } \in \mathbb {Z}^d} \left( 2\mu ^0\Vert \varvec{\xi }\Vert ^2 \hat{\varvec{ u}}(\varvec{\xi }) \,\right. \nonumber \\&\qquad \left. + (\mu ^0 + \lambda ^0) (\varvec{\xi }\cdot \hat{\varvec{ u}}(\varvec{\xi })) \varvec{\xi }\right) \, e^{i \, \varvec{\xi } \cdot \varvec{x}},\nonumber \\&\qquad \varvec{ x}\in Y. \end{aligned}$$

Equating Fourier coefficients of the left-hand and the right-hand side of the equation (1) permits to identify the Fourier coefficients of the displacement-field $\varvec{ u}$ from those of the body-force field $\varvec{ f}$.

If displacement-boundary conditions are considered for the displacement-fluctuation field $\varvec{ u}$ in equation (1), it is natural to represent the fields by sine series

4 $$\begin{aligned} \varvec{ u}(\varvec{ x})&= \sum _{\varvec{k} \in \mathbb {N}^2} \hat{\varvec{ u}}(\textbf{k}) \, \sin (k_1 x_1)\sin (k_2 x_2), \end{aligned}$$

5 $$\begin{aligned} \varvec{ f}(\varvec{ x})&= \sum _{\varvec{k} \in \mathbb {N}^2} \hat{\varvec{ f}}(\textbf{k}) \, \sin (k_1 x_1)\sin (k_2 x_2), \quad \varvec{ x}\in [0,\pi ]^2, \end{aligned}$$

and where we restrict to $d=2$ dimensions for exposition. Following the same strategy as in the periodic case runs into difficulties, however. Inspecting the $(\mu ^0 + \lambda ^0)$-term in Lamé’s operator, we observe

6 $$\begin{aligned} \nabla&\text {div }\varvec{ u}(\varvec{ x}) \nonumber \\&= -\sum _{\varvec{k} \in \mathbb {N}^2} \begin{bmatrix} k_1^2 \hat{u}_1(\textbf{k}) \\ k_2^2 \hat{u}_2(\textbf{k}) \end{bmatrix} \, \sin (k_1 x_1)\sin (k_2 x_2)\nonumber \\&+\sum _{\varvec{k} \in \mathbb {N}^2} k_1k_2 \, \begin{bmatrix} \hat{u}_2(\textbf{k})\\ \hat{u}_1(\textbf{k}) \end{bmatrix} \, \cos (k_1 x_1)\cos (k_2 x_2) \end{aligned}$$

for $\varvec{ x}\in [0,\pi ]^2$, i.e., the appearance of cosine terms is not accounted for in the right-hand side $\varvec{ f}$. For the 3D case, similar (but more complicated) issues arise.

Only in case of a specific mixture of Dirichlet and Neumann boundary conditions [69, 98], more precisely vanishing normal displacement and prescribed tangential components of the normal stress, FFT-based solvers may be developed [32].

As Dirichlet boundary conditions are sometimes required by the application at hand, a suitable workaround needed to be found. Based on the realization that a field with vanishing boundary data on a rectangular cell is periodic for trivial reasons, Dirichlet boundary conditions may be enforced via suitable constraints on a periodic displacement-field. Thus, suitable forces need to be determined which ensure the clamping at the boundary. These forces correspond to Lagrange multipliers in an optimization setting.

A simple way to enforce constraints in optimization is via the penalty method. Translated to the setting at hand, an extremely stiff material may be prescribed to the boundary voxels [3, 4]. Alternatively, the sought forces may be considered as unknown and solved for by suitable solution schemes. Searching for the periodic displacement and the boundary forces jointly, however, leads to an indefinite system of equations, which may either be solved in a staggered fashion [30] or by a Krylov subspace method [72]. Preconditioning indefinite systems, however, is non-trivial, and typically less efficient for indefinite systems than for definite systems [95, §5.2]. A possible work-around consists of eliminating the displacement degrees of freedom and solving for the boundary forces only, see To et al. [90]. This approach, however, appears restricted to linear elastic problems.

### Contributions

The starting point for the work at hand are recent advances [27, 61, 63, 70], which concern computational homogenization problems for thermal conductivity and introduced a unified framework for treating periodic, Dirichlet and Neumann boundary conditions in this setting. In fact, no problems occur when constructing Green’s function for the Laplacian using either sine or cosine series, as differentiating the (co)sine function twice gives a multiple of the (co)sine function one started out with [15, 22, 26]. As mechanical problems are of immediate interest, we turned our attention to this matter.

The underlying idea for the article at hand goes back to the work of Kabel et al. [38], who considered FFT-based solution schemes for finite-strain problems. In this context, the authors [38] noticed that a finite-strain hyperelastic problem could be written in terms of a Lippmann–Schwinger equation which involved either Green’s operator for small strains (1) or Green’s operator for the vector Laplacian $\Delta$. Both strategies turned out to be produce viable and efficient Lippmann–Schwinger solvers.

In the work at hand, we turn this idea upside down: We use Green’s operator for the vector Laplacian, which may be considered as being associated to finite-strains, for problems at small strains. In this way, we circumvent the issue with Green’s operator for small strains and Dirichlet boundary conditions (6).

The price to pay for this idea is a slightly increased iteration count for the Lippmann–Schwinger solvers as a consequence of the misfit in Green’s operator. Moreover, if implemented on the deformation gradient, nine instead of six components need to be treated (both in real and in Fourier space), leading to a certain computational overhead. Yet, the approach, worked out in detail in section 2, avoids Lagrange multipliers and remains in a primal setting. In particular, the entire bouquet of Lippmann–Schwinger technology [48, 79, 88] is readily applicable.

In general, there are two different strategies to develop FFT-based schemes, depending on whether one discretizes first and derives the Lippmann–Schwinger equation afterwards or applies a discretization scheme to the Lippmann–Schwinger equation in a continuous setting [7, 8]. We follow the first route, and revisit the original Moulinec–Suquet discretization [65, 66], which may be interpreted [73, 94] as a non-conforming discretization based on trigonometric polynomials. More precisely, the non-conformity arises from using the trapezoidal rule to approximate the weak form of the balance of linear momentum [81–83]. In section 3, we develop the Moulinec–Suquet discretization in this setting. In fact, we start with truncated sine series and employ the trapezoidal rule for quadrature. In contrast to the periodic case, the boundary points of the cell need to be treated differently in terms of the quadrature for Dirichlet boundary conditions. We develop the corresponding Lippmann–Schwinger equation, operating on the displacement-gradient field, and discuss details of the implementation.

We investigate the computational performance of the novel scheme in section 4 for simple problems and more sophisticated examples, both in terms of the microstructure and the considered constitutive laws.

As a final word of warning, we wish to stress that when referring to “boundary conditions”, we mean the boundary conditions imposed on the displacement fluctuation on the domain edges only. In FFT-based computational micromechanics, it is customary to label the imposed macroscopic strain or stress as a “boundary condition” [5, 40, 51], as well.

## Homogenization with Dirichlet boundary conditions

### The homogenization problem and its variational formulation

We consider a cubic^1^ box $Y=[0,L]^3$, and suppose that a heterogeneous stress operator

7 $$\begin{aligned} {{\,\mathrm{\mathcal {\varvec{S}}}\,}}: Y \times \mathbb {R}^{3 \times 3} \rightarrow \mathbb {R}^{3 \times 3}, \quad (\varvec{ x},\varvec{ F})\mapsto {{\,\mathrm{\mathcal {\varvec{S}}}\,}}(\varvec{ x},\varvec{ F}), \end{aligned}$$

is given, which computes the Cauchy stress response of a deformation gradient $\varvec{ F}$ at the microscopic point $\varvec{ x}\in Y$ and encodes the microstructure under consideration.

Actually, we work at small strains, but choose to work on the deformation gradient $\varvec{ F}$ due to certain computational requirements which will become clear later. To render our investigations mathematically well-defined, we suppose that the stress operator (7) satisfies a number of salient properties.

1. We suppose that the stress ${{\,\mathrm{\mathcal {\varvec{S}}}\,}}(\varvec{ x},\varvec{ F})$ is symmetric for (almost) all $\varvec{ x}\in Y$ and $\varvec{ F}\in \mathbb {R}^{3 \times 3}$, encoding the balance of angular momentum.
2. We assume that the operator ${{\,\mathrm{\mathcal {\varvec{S}}}\,}}$ is uniformly strongly monotone in the sense that there is a positive constant $\alpha _-$, s.t. the inequality
  8 $$\begin{aligned} \left\langle {{\,\mathrm{\mathcal {\varvec{S}}}\,}}(\varvec{ x},\varvec{ F}_1) - {{\,\mathrm{\mathcal {\varvec{S}}}\,}}(\varvec{ x},\varvec{ F}_2) , \varvec{ F}_1 - \varvec{ F}_2 \right\rangle \nonumber \\ \ge \alpha _- \Vert \varvec{\varepsilon }_1 - \varvec{\varepsilon }_2 \Vert ^2 \end{aligned}$$
  holds for all $\varvec{ F}_1,\varvec{ F}_2 \in \mathbb {R}^{3 \times 3}$ and (almost) every $\varvec{ x}\in Y$. Here, the brackets encode the Frobenius inner product
  9 $$\begin{aligned} \langle \varvec{ A},\varvec{ B}\rangle := \text {tr}(\varvec{ B}^T\varvec{ A}), \quad \varvec{ A}, \varvec{ B}\in \mathbb {R}^{3 \times 3}, \end{aligned}$$
  on $3 \times 3$-matrices, $\Vert \varvec{ A}\Vert =\sqrt{\langle \varvec{ A}, \varvec{ A}\rangle }$ refers to the associated norm and the strains
  10 $$\begin{aligned} \varvec{\varepsilon }_i = \frac{1}{2} \left( \varvec{ F}_i + \varvec{ F}_i^T\right) , \quad i=1,2, \end{aligned}$$
  correspond to the symmetric part of the deformation gradient.
3. Moreover, we suppose that the operator ${{\,\mathrm{\mathcal {\varvec{S}}}\,}}$ is uniformly Lipschitz continuous in the sense that there is a positive constant $\alpha _+$, s.t. the inequality
  11 $$\begin{aligned} \Vert {{\,\mathrm{\mathcal {\varvec{S}}}\,}}(\varvec{ x},\varvec{ F}_1) - {{\,\mathrm{\mathcal {\varvec{S}}}\,}}(\varvec{ x},\varvec{ F}_2) \Vert \le \alpha _+ \Vert \varvec{\varepsilon }_1 - \varvec{\varepsilon }_2 \Vert \end{aligned}$$
  holds for all $\varvec{ F}_1,\varvec{ F}_2 \in \mathbb {R}^{3 \times 3}$ and (almost) every $\varvec{ x}\in Y$. The strains $\varvec{\varepsilon }_i$ are defined as in Eq. (10).

The quantity $\kappa = \alpha _+ / \alpha _-$ is called material contrast and determines how hard it is to solve the equilibrium equation we will be considering shortly from a numerical point of view.

We enforce Dirichlet boundary conditions, i.e., for a prescribed macroscopic strain

12 $$\begin{aligned} \bar{\varvec{\varepsilon }} \in \text {Sym}(3) := \left\{ \varvec{ F}\in \mathbb {R}^{3 \times 3} \, \Big | \, \varvec{ F}^T = \varvec{ F}\right\} , \end{aligned}$$

we seek a displacement-fluctuation field

13 $$\begin{aligned} \varvec{ u}&\in {H^1_0(Y;\mathbb {R}^3)}\nonumber \\&= \left\{ \varvec{ u}\in H^1(Y;\mathbb {R}^3) \,\Big |\, \varvec{ u}(\varvec{ x}) = 0 \quad \text {on} \quad \partial Y \right\} \end{aligned}$$

solving the balance of linear momentum (at small strains without inertia and microscopic body forces)

14 $$\begin{aligned} \text {div }{{\,\mathrm{\mathcal {\varvec{S}}}\,}}(\cdot , \bar{\varvec{\varepsilon }} + \nabla \varvec{ u}) = 0, \end{aligned}$$

where $\nabla$ stands for the gradient operator and the divergence operator is the negative of the formal adjoint of the gradient operator, i.e., implicitly characterized by

15 $$\begin{aligned} \varvec{ f}&= \text {div }\varvec{\sigma }\end{aligned}$$

precisely if

16 $$\begin{aligned} \int _Y \varvec{ f}\cdot \varvec{ v}\, d\varvec{ x}&= - \int _Y \langle \varvec{\sigma },\nabla \varvec{ v}\rangle \, d\varvec{ x}\end{aligned}$$

for all

17 $$\begin{aligned} \varvec{ v}&\in H^1_0(Y;\mathbb {R}^3) \end{aligned}$$

for an arbitrary stress field $\varvec{\sigma }\in L^2 (Y;\mathbb {R}^{3 \times 3})$. It is not difficult to show that the problem (14) admits a unique solution $\varvec{ u}\in H^1_0(Y;\mathbb {R}^3)$ under the conditions (11) and (8), see, e.g., Schneider [78, §2]. Then, the apparent stress is defined via averaging

18 $$\begin{aligned} {{\,\mathrm{\mathcal {\varvec{S}}}\,}}^{\text {app}}(\bar{\varvec{\varepsilon }}) = \left\langle { {{\,\mathrm{\mathcal {\varvec{S}}}\,}}(\cdot , \bar{\varvec{\varepsilon }} + \nabla \varvec{ u})} \right\rangle _Y, \end{aligned}$$

where the notation $\left\langle {q} \right\rangle _Y$ computes the mean value of the field *q* over the considered cell

19 $$\begin{aligned} \left\langle {q} \right\rangle _Y = \frac{1}{L^3} \int _Y q(\varvec{ x}) \, d\varvec{ x}. \end{aligned}$$

Here, the term *apparent* is used in Eq. (18) because typically the microstructure arises as a snapshot of a random material [42, 87], and the Dirichlet boundary conditions lead to apparent properties which require a proper infinite-volume limit of the cell to converge to the effective properties under consideration.

By definition of the divergence (15), the displacement field $\varvec{ u}\in H^1_0(Y;\mathbb {R}^3)$ satisfies the equilibrium equation (14) if and only if

20 $$\begin{aligned} \int _Y \langle \nabla \varvec{ v}, {{\,\mathrm{\mathcal {\varvec{S}}}\,}}(\cdot , \bar{\varvec{\varepsilon }} + \nabla \varvec{ u}) \rangle \, d \varvec{ x}= 0 \end{aligned}$$

holds for all

21 $$\begin{aligned} \varvec{ v}\in H^1_0(Y;\mathbb {R}^3). \end{aligned}$$

Typically, the latter formulation (20) is called the *weak formulation* of the problem (14).

### Reformulation via sine series and Green’s operator

With the numerical resolution of the equilibrium problem (20) in mind, we consider a representation of the sought displacement field $\varvec{ u}\in H^1_0(Y;\mathbb {R}^3)$ via a sine series

22 $$\begin{aligned} \varvec{ u}(\varvec{ x}) = \sum _{\textbf{k}\in \mathbb {N}^3} \hat{\varvec{ u}}(\textbf{k}) \, \texttt {sss}(\textbf{k},\varvec{ x}), \quad \varvec{ x}\in Y, \end{aligned}$$

where the convergence is considered in the Sobolev space $H^1_0$ to preserve the Dirichlet boundary conditions. Here, $\mathbb {N}^3$ denotes triples of natural numbers ($\mathbb {N}=\{1,2,3,\ldots \}$), $\hat{\varvec{ u}}(\textbf{k})$ refers to the vector-valued sine coefficient of the field $\varvec{ u}$ at the frequency $\textbf{k}$,

23 $$\begin{aligned} \hat{\varvec{ u}}(\textbf{k}) = \frac{8}{L^3} \int _Y \varvec{ u}(\varvec{ x}) \texttt {sss}(\textbf{k}, \varvec{ x}) \, d\varvec{ x}, \quad \textbf{k}\in \mathbb {N}^3, \end{aligned}$$

and we use the shorthand notation

24 $$\begin{aligned}&\texttt {sss}(\textbf{k},\varvec{ x}) \nonumber \\&=\sin \left( \frac{k_1 \pi x_1}{L}\right) \sin \left( \frac{k_2 \pi x_2}{L}\right) \sin \left( \frac{k_3 \pi x_3}{L}\right) \end{aligned}$$

for

25 $$\begin{aligned} \textbf{k}&=(k_1,k_2,k_3) \in \mathbb {N}^3, \quad \varvec{ x}\in Y. \end{aligned}$$

The deformation gradient of the field (22) computes as

26 $$\begin{aligned}&\nabla \varvec{ u}(\varvec{ x}) = \frac{\pi }{L}\sum _{\textbf{k}\in \mathbb {N}^3}\nonumber \\&\quad \begin{bmatrix} k_1\texttt {css}(\textbf{k},\varvec{ x}) &{} k_1\texttt {css}(\textbf{k},\varvec{ x}) &{} k_1 \texttt {css}(\textbf{k},\varvec{ x}) \\ k_2\texttt {scs}(\textbf{k},\varvec{ x}) &{} k_2\texttt {scs}(\textbf{k},\varvec{ x}) &{} k_2 \texttt {scs}(\textbf{k},\varvec{ x}) \\ k_3\texttt {ssc}(\textbf{k},\varvec{ x}) &{} k_3\texttt {ssc}(\textbf{k},\varvec{ x}) &{} k_3 \texttt {ssc}(\textbf{k},\varvec{ x}) \\ \end{bmatrix} \begin{bmatrix} \hat{u}_1(\textbf{k})\\ \hat{u}_2(\textbf{k})\\ \hat{u}_3(\textbf{k}) \end{bmatrix},\nonumber \\&\quad \varvec{ x}\in Y, \end{aligned}$$

where $\hat{u}_j$ refers to the sine coefficients of the *j*-th component of the displacement field $\varvec{ u}$. We suppose that a “reference material” $\mathbb {C}^0$ is given which acts via

27 $$\begin{aligned} \mathbb {C}^0 : \varvec{ F}= \alpha ^0 \varvec{ F}\end{aligned}$$

on a deformation gradient

28 $$\begin{aligned} \varvec{ F}\in \mathbb {R}^{3 \times 3}. \end{aligned}$$

Notice that such a material is non-physical, but is chosen for numerical purposes. We refer to Kabel et al. [38] for a related study in the finite-strain setting. For a reference material of the type (27), we wish to determine Green’s operator $\varvec{ G}^0 \in L(H^{-1}_0(Y;\mathbb {R}^3),H^{1}_0(Y;\mathbb {R}^3))$, where $H^{-1}_0(Y;\mathbb {R}^3)$ denotes the continuous dual space of the Banach space $H^{1}_0(Y;\mathbb {R}^3)$. Green’s operator $\varvec{ G}^0$ is implicitly characterized by the condition

29 $$\begin{aligned} \varvec{ u}= \varvec{ G}^0 \varvec{ f}\quad \text {if and only if} \quad \text {div }\mathbb {C}^0:\nabla \varvec{ u}= \varvec{ f}\end{aligned}$$

for all $\varvec{ f}\in H^{-1}_0(Y;\mathbb {R}^3)$ and $\varvec{ u}\in H^1_0(Y;\mathbb {R}^3)$. By definition of the divergence operator (15), the latter condition may also be re-written in weak form

30 $$\begin{aligned} \int _Y&\langle \nabla \varvec{ v},\mathbb {C}^0:\nabla \varvec{ u}\rangle \, d\varvec{ x}\nonumber \\&= - \int _Y \varvec{ v}\cdot \varvec{ f}\, d\varvec{ x}\quad \text {for all} \quad \varvec{ v}\in H^1_0(Y;\mathbb {R}^3). \end{aligned}$$

To evaluate the condition (30) explicitly, we expand the fields $\varvec{ f}$ and $\varvec{ v}$ in sine series (22),

31 $$\begin{aligned} \varvec{ f}(\varvec{ x})&= \sum _{\textbf{k}\in \mathbb {N}^3} \hat{\varvec{ f}}(\textbf{k}) \, \texttt {sss}(\textbf{k},\varvec{ x}) \end{aligned}$$

and

32 $$\begin{aligned} \varvec{ v}(\varvec{ x})&= \sum _{\varvec{ l}\in \mathbb {N}^3} \hat{\varvec{ v}}(\varvec{ l}) \, \texttt {sss}(\varvec{ l},\varvec{ x}) \end{aligned}$$

for $\varvec{ x}\in Y$, respectively, with corresponding sine coefficients. Then, the right-hand side of the condition (30) becomes

33 $$\begin{aligned} - \int _Y \varvec{ v}\cdot \varvec{ f}\, d\varvec{ x}= - \frac{L^3}{8}\sum _{\textbf{k}\in \mathbb {N}^3} \hat{\varvec{ v}}(\textbf{k}) \cdot \hat{\varvec{ f}}(\textbf{k}), \end{aligned}$$

where we used the first of the (elementary) integral identities

34 $$\begin{aligned} \begin{aligned} \int _0^L \sin \left( \frac{k \pi x}{L} \right) \sin \left( \frac{\ell \pi x}{L} \right) \, dx&= \frac{L}{2} \, \delta _{k \ell }, \\ \int _0^L \cos \left( \frac{k \pi x}{L} \right) \cos \left( \frac{\ell \pi x}{L} \right) \, dx&= \frac{L}{2} \, \delta _{k \ell }, \\ \end{aligned} \end{aligned}$$

valid for $k,\ell \in \mathbb {N}$, for each individual coordinate.

With the help of the representation (26) for the field $\varvec{ v}$ and the integral identities (34), we obtain

35 $$\begin{aligned} \int _Y&\langle \nabla \varvec{ v},\mathbb {C}^0:\nabla \varvec{ u}\rangle \, d\varvec{ x}\nonumber \\&= {\alpha ^0\frac{\pi ^2L}{8}\sum _{\textbf{k}\in \mathbb {N}^3}} \Vert \textbf{k}\Vert ^2 \, \hat{\varvec{ v}}(\textbf{k}) \cdot \hat{\varvec{ u}}(\textbf{k}). \end{aligned}$$

Comparing coefficients with the left-hand side (33) yields the formula

36 $$\begin{aligned} \hat{\varvec{ f}}(\textbf{k}) = \widehat{\varvec{ G}}^0(\textbf{k})\, \hat{\varvec{ u}}(\textbf{k}), \quad \textbf{k}\in \mathbb {N}^3 \end{aligned}$$

with the $3 \times 3$ matrix

37 $$\begin{aligned} \widehat{\varvec{ G}}^0(\textbf{k}) = - \frac{L^2}{\alpha ^0 \pi ^2 \Vert \textbf{k}\Vert ^2} \,\varvec{ I}\end{aligned}$$

for Green’s operator in the sine-series representation (22). With Green’s operator at hand, the Lippmann–Schwinger equation

38 $$\begin{aligned} \varvec{ F}+ \Gamma ^0 : ({{\,\mathrm{\mathcal {\varvec{S}}}\,}}(\cdot , \varvec{ F}) - \mathbb {C}^0:\varvec{ F}) = \bar{\varvec{\varepsilon }}, \quad \Gamma ^0 = \nabla \varvec{ G}^0 \text {div }\end{aligned}$$

for the displacement-gradient field $\varvec{ F}\in L^2 (Y;\mathbb {R}^{3 \times 3})$ associated to the equation (14) is readily derived and shown to be equivalent to the original formulation by the standard procedure, see, e.g., Kabel et al. [38].

## The Moulinec–Suquet discretization with Dirichlet boundary conditions

### Ansatz space and equilibrium equation

**Fig. 1:**
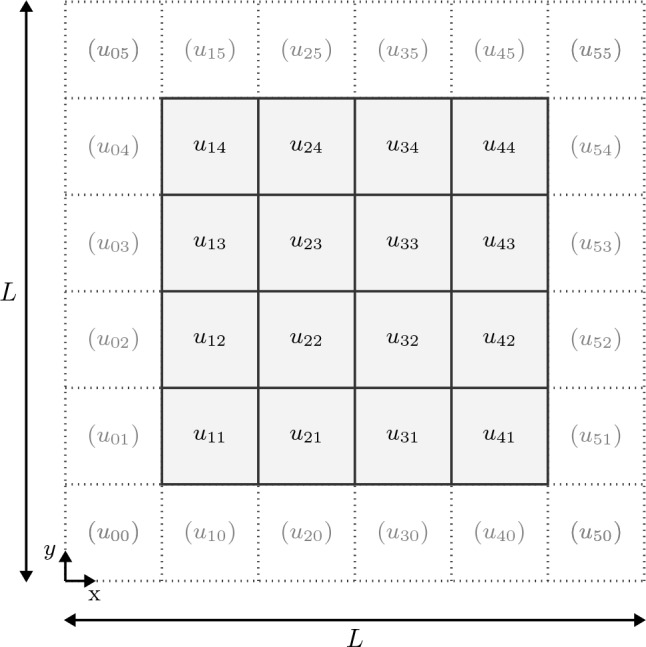
Two-dimensional sketch of a $6\times 6$-pixel grid with displacement values $u_{ij}$ for $i,j=0,1, \ldots , 5$. Dirichlet boundary conditions are enforced at the pixels in brackets

As our discretization, we work with a truncation of the sine series (22), i.e., we consider an ansatz for the displacement-fluctuation field in terms of a sine polynomial

39 $$\begin{aligned} \varvec{ u}(\varvec{ x}) = \sum _{\textbf{k}\in \mathbb {N}_N^3} \hat{\varvec{ u}}(\textbf{k}) \, \texttt {sss}(\textbf{k},\varvec{ x}), \quad \varvec{ x}\in Y, \end{aligned}$$

for $\mathbb {N}_N = \{1,2,\ldots ,N-2\}$ where $N \ge 3$ is an integer that will serve as the voxel count per axis in the succeeding. We will write $\texttt {S}_N(Y;\mathbb {R}^3)$ for the space of such sine polynomials. By construction, the space $\texttt {S}_N(Y;\mathbb {R}^3)$ of sine polynomials is a proper subset of the Sobolev space $H^1_0(Y;\mathbb {R}^3)$.

We define the discrete grid - interpreted as the *voxel* grid later on -

40 $$\begin{aligned} Y_N&\left\{ \varvec{ x}_{\textbf{j}} = \left( \frac{L\, j_1}{N-1}, \frac{L\, j_2}{N-1}, \frac{L\, j_3}{N-1}\right) \in Y \right. {}\nonumber \\&\,\,\left. \Big | \,\, {\textbf{j}}=(j_1,j_2,j_3) \in \{0,1,\ldots ,N-1\}^3\right\} \end{aligned}$$

which serves two purposes. For a start, the values at the grid points (40), illustrated in Fig. 1, comprise the real counterpart of the field (39) and its gradient

41 $$\begin{aligned}&\nabla \varvec{ u}(\varvec{ x}) = \frac{\pi }{L}\sum _{\textbf{k}\in \mathbb {N}_N^3}\nonumber \\&\quad \begin{bmatrix} k_1\texttt {css}(\textbf{k},\varvec{ x}) &{} k_1\texttt {css}(\textbf{k},\varvec{ x}) &{} k_1 \texttt {css}(\textbf{k},\varvec{ x}) \\ k_2\texttt {scs}(\textbf{k},\varvec{ x}) &{} k_2\texttt {scs}(\textbf{k},\varvec{ x}) &{} k_2 \texttt {scs}(\textbf{k},\varvec{ x}) \\ k_3\texttt {ssc}(\textbf{k},\varvec{ x}) &{} k_3\texttt {ssc}(\textbf{k},\varvec{ x}) &{} k_3 \texttt {ssc}(\textbf{k},\varvec{ x}) \\ \end{bmatrix} \begin{bmatrix} \hat{u}_1(\textbf{k})\\ \hat{u}_2(\textbf{k})\\ \hat{u}_3(\textbf{k}) \end{bmatrix},\nonumber \\&\quad \varvec{ x}\in Y, \end{aligned}$$

The translation between the representation (39) in “Fourier” space and on the grid (40) in real space is handled via discrete sine/cosine transforms, see section 3.4.

The second purpose of the grid (40) is to provide $N^3$ integration points which are used to approximate the weak form (20) of the equilibrium equation. We use a quadrature rule which permits to integrate the terms appearing in the gradient (26) of the truncated field (39) exactly. More precisely, for the weights

42 $$\begin{aligned} w_j = \left\{ \begin{array}{rl} \frac{1}{2(N-1)}, &{}j \in \{0,N-1\},\\ \frac{1}{N-1}, &{}j = 1,2,\ldots ,N-2,\\ \end{array} \right. \end{aligned}$$

the identity

43 $$\begin{aligned} \frac{1}{L}&\int _0^L \phi (x)\psi (x) \, dx \nonumber \\&= \sum _{j=0}^{N-1} w_j \, \phi \left( \frac{L j}{N-1} \right) \psi \left( \frac{L j}{N-1} \right) \end{aligned}$$

holds for the sine polynomials

$$\begin{aligned} \phi (x)&= \sum _{k=1}^{N-2} \hat{\phi }_k \sin \left( \frac{\pi }{L}\, kx\right) \end{aligned}$$

and

44 $$\begin{aligned} \psi (x)&= \sum _{\ell =1}^{N-2} \hat{\psi }_\ell \sin \left( \frac{\pi }{L}\, \ell x\right) \end{aligned}$$

with sine coefficients $\hat{\phi }_k$ and $\hat{\psi }_\ell$. Moreover, the identity (43) is also valid for the cosine polynomials

$$\begin{aligned} \phi (x)&= \sum _{k=1}^{N-2} \hat{\phi }_k \cos \left( \frac{\pi }{L}\, kx\right) \end{aligned}$$

and

45 $$\begin{aligned} \psi (x)&= \sum _{\ell =1}^{N-2} \hat{\psi }_\ell \cos \left( \frac{\pi }{L}\, \ell x\right) \end{aligned}$$

with cosine coefficients $\hat{\phi }_k$ and $\hat{\psi }_\ell$. The latter also satisfy the following identity

46 $$\begin{aligned} \sum _{j=0}^{N-1} w_j \, \phi \left( \frac{L j}{N-1} \right) = 0, \end{aligned}$$

which ensures that the mean value of the cosine polynomial (45) (which is zero) is properly computed by the trapezoidal rule (42). Notice that the considered cosine polynomials (45) correspond to those functions which arise as derivatives of the sine polynomials (44) - in particular, no constant term appears.

For the cosine polynomials (45), the two boundary points need to be considered with half the weight of the interior points in the quadrature rule (43). The sine polynomials (44) vanish at these boundary points, anyway, so the quadrature weights at the boundary do no influence the accuracy of the rule in this case. Rather, the rule (43) is chosen to cover both considered cases simultaneously.

With the one-dimensional quadrature rules (43) and (46) at hand, the specific form of the grid (40) and the gradient field (41) implies the formulas

47 $$\begin{aligned} \frac{1}{L^3} \int _Y \langle \nabla \varvec{ u}, \nabla \varvec{ v}\rangle \, d\varvec{ x}= \sum _{\varvec{ x}_{\textbf{j}} \in Y_N} w_{\textbf{j}} \, \langle \nabla \varvec{ u}(\varvec{ x}_{\textbf{j}}) , \nabla \varvec{ v}(\varvec{ x}_{\textbf{j}}) \rangle \end{aligned}$$

and

48 $$\begin{aligned} \sum _{\varvec{ x}_{\textbf{j}} \in Y_N} w_{\textbf{j}} \, \nabla \varvec{ u}(\varvec{ x}_{\textbf{j}}) = 0 \end{aligned}$$

with weights $w_{\textbf{j}} = w_{j_1} w_{j_2} w_{j_3}$, valid for all fields $\varvec{ u}, \varvec{ v}\in \texttt {S}_N(Y;\mathbb {R}^3)$. The identity (47) provides an exact quadrature formula for bilinear expressions in gradients of sine polynomials (39). With this identity at hand, we proceed to define a natural discrete equivalent of the equilibrium equation (14) (in weak form (20)). For a given non-linear stress operator ${{\,\mathrm{\mathcal {\varvec{S}}}\,}}$ and prescribed macroscopic strain $\bar{\varvec{\varepsilon }}$, we seek a displacement-fluctuation field $\varvec{ u}\in \texttt {S}_N(Y;\mathbb {R}^3)$, s.t. the equation

49 $$\begin{aligned} \sum _{\varvec{ x}_{\textbf{j}} \in Y_N} w_{\textbf{j}} \, \langle \nabla \varvec{ v}(\varvec{ x}_{\textbf{j}}), {{\,\mathrm{\mathcal {\varvec{S}}}\,}}(\varvec{ x}_{\textbf{j}}, \bar{\varvec{\varepsilon }} + \nabla \varvec{ u}(\varvec{ x}_{\textbf{j}}))\rangle = 0 \end{aligned}$$

holds for all

50 $$\begin{aligned}&\varvec{ v}\in \texttt {S}_N(Y;\mathbb {R}^3). \end{aligned}$$

With the quadrature formula (47) at hand, existence and uniqueness of solutions to the discrete problem (49) are readily established, see, e.g., Schneider [73, 82] for the techniques to be used.

### Lippmann–Schwinger reformulation

To arrive at a discrete equivalent of the Lippmann–Schwinger equation (38)

51 $$\begin{aligned} \varvec{ F}+ \Gamma ^0 : ({{\,\mathrm{\mathcal {\varvec{S}}}\,}}(\cdot , \varvec{ F}) - \mathbb {C}^0:\varvec{ F}) = \bar{\varvec{\varepsilon }}, \quad \Gamma ^0 = \nabla \varvec{ G}^0 \text {div }, \end{aligned}$$

we introduce the (finite-dimensional) vector space

52 $$\begin{aligned} L^2(Y_N;\mathbb {R}^{3 \times 3}) = \left\{ \varvec{\tau }: Y_N \rightarrow \mathbb {R}^{3 \times 3}\right\} \end{aligned}$$

of discrete tensor fields on the discrete grid (40). Endowed with the inner product

53 $$\begin{aligned} \left\langle \varvec{\tau }_1, \varvec{\tau }_2 \right\rangle _{L^2}&:= \sum _{\varvec{ x}_{\textbf{j}} \in Y_N} w_{\textbf{j}} \, \langle \varvec{\tau }_1(\varvec{ x}_{\textbf{j}}), \varvec{\tau }_2(\varvec{ x}_{\textbf{j}})\rangle , \nonumber \\&\quad \varvec{\tau }_1,\varvec{\tau }_2 \in L^2(Y_N;\mathbb {R}^{3 \times 3}), \end{aligned}$$

the space $L^2(Y_N;\mathbb {R}^{3 \times 3})$ becomes a (finite-dimensional) Hilbert space. With this notation at hand, we introduce the discrete gradient operator

54 $$\begin{aligned} \nabla _N : \texttt {S}_N(Y;\mathbb {R}^3) \rightarrow L^2(Y_N;\mathbb {R}^{3 \times 3}), \end{aligned}$$

acting on sine polynomials (39) via point evaluation, i.e.,

55 $$\begin{aligned} \left( \nabla _N \varvec{ u}\right) (\varvec{ x}_{\textbf{j}}) = \nabla \varvec{ u}(\varvec{ x}_{\textbf{j}}). \end{aligned}$$

Due to the representation (39)

56 $$\begin{aligned} \varvec{ u}(\varvec{ x}) = \sum _{\textbf{k}\in \mathbb {N}_N^3} \hat{\varvec{ u}}(\textbf{k}) \, \texttt {sss}(\textbf{k},\varvec{ x}), \quad \varvec{ x}\in Y, \end{aligned}$$

and the formula (41)

57 $$\begin{aligned}&\nabla \varvec{ u}(\varvec{ x}) = \frac{\pi }{L}\sum _{\textbf{k}\in \mathbb {N}_N^3}\nonumber \\&\quad \begin{bmatrix} k_1\texttt {css}(\textbf{k},\varvec{ x}) &{} k_1\texttt {css}(\textbf{k},\varvec{ x}) &{} k_1 \texttt {css}(\textbf{k},\varvec{ x}) \\ k_2\texttt {scs}(\textbf{k},\varvec{ x}) &{} k_2\texttt {scs}(\textbf{k},\varvec{ x}) &{} k_2 \texttt {scs}(\textbf{k},\varvec{ x}) \\ k_3\texttt {ssc}(\textbf{k},\varvec{ x}) &{} k_3\texttt {ssc}(\textbf{k},\varvec{ x}) &{} k_3 \texttt {ssc}(\textbf{k},\varvec{ x}) \\ \end{bmatrix} \begin{bmatrix} \hat{u}_1(\textbf{k})\\ \hat{u}_2(\textbf{k})\\ \hat{u}_3(\textbf{k}) \end{bmatrix},\nonumber \\&\quad \varvec{ x}\in Y, \end{aligned}$$

we obtain

58 $$\begin{aligned}&\nabla _N \varvec{ u}(\varvec{ x}_{\textbf{j}}) = \frac{\pi }{L}\sum _{\textbf{k}\in \mathbb {N}_N^3}\nonumber \\&\quad \begin{bmatrix} k_1\texttt {css}(\textbf{k},\varvec{ x}_{\textbf{j}}) &{} k_1\texttt {css}(\textbf{k},\varvec{ x}_{\textbf{j}}) &{} k_1\texttt {css}(\textbf{k},\varvec{ x}_{\textbf{j}}) \\ k_2\texttt {scs}(\textbf{k},\varvec{ x}_{\textbf{j}}) &{} k_2\texttt {scs}(\textbf{k},\varvec{ x}_{\textbf{j}}) &{} k_2\texttt {scs}(\textbf{k},\varvec{ x}_{\textbf{j}}) \\ k_3\texttt {ssc}(\textbf{k},\varvec{ x}_{\textbf{j}}) &{} k_3\texttt {ssc}(\textbf{k},\varvec{ x}_{\textbf{j}}) &{} k_3\texttt {ssc}(\textbf{k},\varvec{ x}_{\textbf{j}}) \\ \end{bmatrix} \begin{bmatrix} \hat{u}_1(\textbf{k})\\ \hat{u}_2(\textbf{k})\\ \hat{u}_3(\textbf{k}) \end{bmatrix}. \end{aligned}$$

Inspecting the terms via the shorthand notation (24), e.g.,

59 $$\begin{aligned}&\texttt {css}(\textbf{k},\varvec{ x}_{\textbf{j}})\nonumber \\&\quad = \cos \left( \frac{\pi k_1 j_1}{N-1}\right) \sin \left( \frac{\pi k_2 j_2}{N-1}\right) \sin \left( \frac{ \pi k_3 j_3}{N-1}\right) , \end{aligned}$$

we realize that the sums appearing in equation (58) actually correspond to discrete sine and cosine transforms, a fact that we will further look into in section 3.4.

In addition to the discrete version of the gradient operator (54), we will also consider a discrete version of the divergence operator (15)

60 $$\begin{aligned} \text {div}_N\,: L^2(Y_N;\mathbb {R}^{3 \times 3}) \rightarrow \texttt {S}_N(Y;\mathbb {R}^3) \end{aligned}$$

as the negative of the adjoint operator to the discrete gradient operator (54). More precisely, for $\varvec{\tau }\in L^2(Y_N;\mathbb {R}^{3 \times 3})$ we postulate

61 $$\begin{aligned} \frac{1}{L^3} \int _Y \text {div}_N\,\varvec{\tau }&\cdot \varvec{ v}\, d\varvec{ x}= - \langle \varvec{\tau }, \nabla _N \varvec{ v}\rangle _{L^2} \end{aligned}$$

to hold for all

62 $$\begin{aligned}&\varvec{ v}\in \texttt {S}_N(Y;\mathbb {R}^3). \end{aligned}$$

To determine an explicit formula for the divergence $\text {div}_N\,$, we fix $\varvec{\tau }\in L^2(Y_N;\mathbb {R}^{3 \times 3})$ and write

63 $$\begin{aligned}&\text {div}_N\,\varvec{\tau }\equiv \varvec{ f}\in \texttt {S}_N(Y;\mathbb {R}^3), \end{aligned}$$

i.e.,

64 $$\begin{aligned}&\varvec{ f}(\varvec{ x}) = \sum _{\textbf{k}\in \mathbb {N}_{N}^3} \hat{\varvec{ f}}(\textbf{k}) \, \texttt {sss}(\textbf{k},\varvec{ x}) \end{aligned}$$

for suitable coefficients $\hat{\varvec{ f}}(\textbf{k})$. Expanding the test field

65 $$\begin{aligned} \varvec{ v}(\varvec{ x}) = \sum _{\textbf{k}\in \mathbb {N}_N^3} \hat{\varvec{ v}}(\textbf{k}) \, \texttt {sss}(\textbf{k},\varvec{ x}) \end{aligned}$$

in the definition (61), using the first of the integral identities (34) and accounting for the definition (53), we arrive at the formula

66 $$\begin{aligned} \frac{1}{8} \sum _{\textbf{k}\in \mathbb {N}_N^3} \hat{\varvec{ f}}(\textbf{k}) \cdot \hat{\varvec{ v}}(\textbf{k}) = - \sum _{\varvec{ x}_{\textbf{j}} \in Y_N} w_{\textbf{j}} \, \varvec{\tau }(\varvec{ x}_{\textbf{j}}):\nabla \varvec{ v}(\varvec{ x}_{\textbf{j}}), \end{aligned}$$

or, in component notation

67 $$\begin{aligned}&\sum _{b=1}^3 \sum _{\textbf{k}\in \mathbb {N}_N^3} \hat{f}_b(\textbf{k}) \hat{v}_b(\textbf{k})\nonumber \\&\quad = - 8\sum _{b=1}^3\sum _{\varvec{ x}_{\textbf{j}} \in Y_N} w_{\textbf{j}} \, \sum _{a=1}^3\tau _{ab}(\varvec{ x}_{\textbf{j}}) \partial _a v_b(\varvec{ x}_{\textbf{j}}). \end{aligned}$$

Differentiating the explicit formula, exploiting the shorthand notation (24),

68 $$\begin{aligned} v_b(\varvec{ x})&= \sum _{\textbf{k}\in \mathbb {N}_N^3} \hat{v}_b(\textbf{k}) \, \texttt {sss}(\textbf{k},\varvec{ x})\nonumber \\&\equiv \sum _{\textbf{k}\in \mathbb {N}_N^3} \hat{v}_b(\textbf{k}) \, \sin \left( \frac{k_1 \pi x_1}{L}\right) \nonumber \\&\quad \cdot \sin \left( \frac{k_2 \pi x_2}{L}\right) \sin \left( \frac{k_3 \pi x_3}{L}\right) , \end{aligned}$$

we arrive at the formula

$$\begin{aligned} \begin{aligned} \partial _1 v_b(\varvec{ x})&\equiv \sum _{\textbf{k}\in \mathbb {N}_N^3} \frac{k_1 \pi }{L} \hat{v}_b(\textbf{k}) \, \cos \left( \frac{k_1 \pi x_1}{L}\right) \\&\quad \cdot \sin \left( \frac{k_2 \pi x_2}{L}\right) \sin \left( \frac{k_3 \pi x_3}{L}\right) \\&= \sum _{\textbf{k}\in \mathbb {N}_N^3} \frac{k_1 \pi }{L}\hat{v}_b(\textbf{k}) \, \texttt {css}(\textbf{k},\varvec{ x}) \end{aligned} \end{aligned}$$

as well as

69 $$\begin{aligned} \begin{aligned} \partial _2 v_b(\varvec{ x})&= \sum _{\textbf{k}\in \mathbb {N}_N^3} \frac{k_2 \pi }{L}\hat{v}_b(\textbf{k}) \, \texttt {scs}(\textbf{k},\varvec{ x}), \\ \partial _3 v_b(\varvec{ x})&= \sum _{\textbf{k}\in \mathbb {N}_N^3} \frac{k_3 \pi }{L}\hat{v}_b(\textbf{k}) \, \texttt {ssc}(\textbf{k},\varvec{ x}). \\ \end{aligned} \end{aligned}$$

Thus, for $b=1,2,3$, we obtain

$$\begin{aligned} \begin{aligned}&\sum _{\varvec{ x}_{\textbf{j}} \in Y_N} w_{\textbf{j}} \, \sum _{a=1}^3\tau _{ab}(\varvec{ x}_{\textbf{j}})\partial _a v_b(\varvec{ x}_{\textbf{j}})\\&\quad = \sum _{\varvec{ x}_{\textbf{j}} \in Y_N} w_{\textbf{j}} \, \left[ \tau _{1b}(\varvec{ x}_{\textbf{j}})\partial _1 v_b(\varvec{ x}_{\textbf{j}})\right. \\&\quad \left. + \tau _{2b}(\varvec{ x}_{\textbf{j}})\partial _2 v_b(\varvec{ x}_{\textbf{j}}) + \tau _{3b}(\varvec{ x}_{\textbf{j}})\partial _3 v_b(\varvec{ x}_{\textbf{j}})\right] \\&\quad = \sum _{\textbf{k}\in \mathbb {N}_N^3} \hat{v}_b(\textbf{k}) \frac{\pi }{L} \sum _{\varvec{ x}_{\textbf{j}} \in Y_N} w_{\textbf{j}} \, \left[ k_1 \tau _{1b}(\varvec{ x}_{\textbf{j}}) \, \texttt {css}(\textbf{k},\varvec{ x}_{\textbf{j}})\right. \nonumber \\&\quad \left. + k_2 \tau _{2b}(\varvec{ x}_{\textbf{j}}) \, \texttt {scs}(\textbf{k},\varvec{ x}_{\textbf{j}}) + k_3 \tau _{3b}(\varvec{ x}_{\textbf{j}}) \, \texttt {ssc}(\textbf{k},\varvec{ x}_{\textbf{j}})\right] . \\ \end{aligned} \end{aligned}$$

As the coefficients $\hat{v}_b(\textbf{k})$ can be chosen arbitrarily and we deduce, by definition (63), the expression

70 $$\begin{aligned} {\hat{f}_b(\textbf{k})=(\widehat{\text {div}_N\,\varvec{\tau }})_b(\textbf{k}),} \end{aligned}$$

the equation (67) implies the explicit formula

71 $$\begin{aligned}&(\widehat{\text {div}_N\,\varvec{\tau }})_b(\textbf{k}) \nonumber \\&\quad {=} -\frac{\pi }{8L} \sum _{\varvec{ x}_{\textbf{j}} \in Y_N} w_{\textbf{j}} \, \left[ k_1 \tau _{1b}(\varvec{ x}_{\textbf{j}}) \, \texttt {css}(\textbf{k},\varvec{ x}_{\textbf{j}})\right. \nonumber \\&\quad + k_2 \tau _{2b}(\varvec{ x}_{\textbf{j}}) \, \texttt {scs}(\textbf{k},\varvec{ x}_{\textbf{j}})\nonumber \\&\quad \left. + k_3 \tau _{3b}(\varvec{ x}_{\textbf{j}}) \, \texttt {ssc}(\textbf{k},\varvec{ x}_{\textbf{j}})\right] \end{aligned}$$

for every component $b=1,2,3$ of the sine coefficients of the discrete divergence operator applied to the field $\varvec{\tau }\in L^2(Y_N;\mathbb {R}^{3 \times 3})$. The formula (71) may also be written in the form

72 $$\begin{aligned} \begin{aligned} (\widehat{\text {div}_N\,\varvec{\tau }})_b(\textbf{k})&= -\frac{k_1 \pi }{8L} \sum _{\varvec{ x}_{\textbf{j}} \in Y_N} w_{\textbf{j}} \,\tau _{1b}(\varvec{ x}_{\textbf{j}}) \, \texttt {css}(\textbf{k},\varvec{ x}_{\textbf{j}})\\&\quad -\frac{k_2\pi }{8L} \sum _{\varvec{ x}_{\textbf{j}} \in Y_N} w_{\textbf{j}} \, \tau _{2b}(\varvec{ x}_{\textbf{j}}) \, \texttt {scs}(\textbf{k},\varvec{ x}_{\textbf{j}})\\&\quad -\frac{k_3\pi }{8L} \sum _{\varvec{ x}_{\textbf{j}} \in Y_N} w_{\textbf{j}} \, \tau _{3b}(\varvec{ x}_{\textbf{j}}) \, \texttt {ssc}(\textbf{k},\varvec{ x}_{\textbf{j}}), \end{aligned} \end{aligned}$$

highlighting the individual discrete sine/cosine transformations that need to be applied, see section (3.4) for more details.

With the definitions (55) and (61) of the discrete derivative operators at hand, we observe that a displacement-fluctuation field $\varvec{ u}\in \texttt {S}_N(Y;\mathbb {R}^3)$ solves the equilibrium equation (49), i.e.,

73 $$\begin{aligned} \sum _{\varvec{ x}_{\textbf{j}} \in Y_N} w_{\textbf{j}} \, \langle \nabla \varvec{ v}(\varvec{ x}_{\textbf{j}}), {{\,\mathrm{\mathcal {\varvec{S}}}\,}}(\varvec{ x}_{\textbf{j}}, \bar{\varvec{\varepsilon }} + \nabla \varvec{ u}(\varvec{ x}_{\textbf{j}}))\rangle = 0 \end{aligned}$$

for all

74 $$\begin{aligned} \varvec{ v}\in \texttt {S}_N(Y;\mathbb {R}^3), \end{aligned}$$

if and only if it solves the operator-type equilibrium equation

75 $$\begin{aligned} \text {div}_N\,{{\,\mathrm{\mathcal {\varvec{S}}}\,}}(\cdot , \bar{\varvec{\varepsilon }} + \nabla _N \varvec{ u}) = 0. \end{aligned}$$

Introducing a reference material $\mathbb {C}^0$, the latter equation may be equivalently rewritten in the form

76 $$\begin{aligned} \text {div}_N\,\mathbb {C}^0: (\bar{\varvec{\varepsilon }} + \nabla _N \varvec{ u}) =&- \text {div}_N\,\left[ {{\,\mathrm{\mathcal {\varvec{S}}}\,}}(\cdot , \bar{\varvec{\varepsilon }} + \nabla _N \varvec{ u}) \right. \nonumber \\&\left. - \mathbb {C}^0:(\bar{\varvec{\varepsilon }} + \nabla _N \varvec{ u})\right] . \end{aligned}$$

Notice that the identity

77 $$\begin{aligned} \text {div}_N\,\mathbb {C}^0: \bar{\varvec{\varepsilon }} = 0 \end{aligned}$$

holds. In fact, by definition (61)

78 $$\begin{aligned} -\langle \text {div}_N\,\mathbb {C}^0: \bar{\varvec{\varepsilon }}, \nabla \varvec{ v}\rangle _{L^2}&=\sum _{\varvec{ x}_{\textbf{j}} \in Y_N} w_{\textbf{j}} \, \langle \mathbb {C}^0 : \bar{\varvec{\varepsilon }},\nabla \varvec{ v}(\varvec{ x}_{\textbf{j}})\rangle \nonumber \\&=\!\left\langle \mathbb {C}^0 : \bar{\varvec{\varepsilon }}, \sum _{\varvec{ x}_{\textbf{j}} \in Y_N}\!w_{\textbf{j}} \, \nabla \varvec{ v}(\varvec{ x}_{\textbf{j}}) \right\rangle \nonumber \\&=0 \end{aligned}$$

holds for all $\varvec{ v}\in \texttt {S}_N(Y;\mathbb {R}^3)$. Here, the second quadrature identity (46) is crucial. Thus, we arrive at the equation

79 $$\begin{aligned}&\text {div}_N\,\mathbb {C}^0: \nabla _N \varvec{ u}\nonumber \\&\quad = - \text {div}_N\,\left[ {{\,\mathrm{\mathcal {\varvec{S}}}\,}}(\cdot , \bar{\varvec{\varepsilon }} + \nabla _N \varvec{ u}) - \mathbb {C}^0:(\bar{\varvec{\varepsilon }} + \nabla _N \varvec{ u})\right] . \end{aligned}$$

By construction of the discrete divergence and the first quadrature identity (43), we may use Green’s operator (29) for the left-hand side to write

80 $$\begin{aligned} \varvec{ u}&= - \varvec{ G}^0 \text {div}_N\,\left[ {{\,\mathrm{\mathcal {\varvec{S}}}\,}}(\cdot , \bar{\varvec{\varepsilon }} + \nabla _N \varvec{ u}) \right. \nonumber \\&\quad \left. - \mathbb {C}^0:(\bar{\varvec{\varepsilon }} + \nabla _N \varvec{ u}))\right] . \end{aligned}$$

Differentiating and adding the macroscopic strain $\bar{\varvec{\varepsilon }}$, we finally arrive at the Lippmann–Schwinger equation

81 $$\begin{aligned} \varvec{ F}&= \bar{\varvec{\varepsilon }} - \Gamma ^0_N : \left[ {{\,\mathrm{\mathcal {\varvec{S}}}\,}}(\cdot , \varvec{ F})-\mathbb {C}^0: \varvec{ F}\right] , \nonumber \\ \quad \Gamma ^0_N&= \nabla _N \varvec{ G}^0 \text {div}_N\,\end{aligned}$$

for the field $\varvec{ F}= \bar{\varvec{\varepsilon }} + \nabla _N \varvec{ u}\in L^2(Y_N;\mathbb {R}^{3 \times 3})$. By standard arguments [73, 82], it can be shown that solutions to the equilibrium equation (75) and the Lippmann–Schwinger equation (81) are strictly equivalent.

### Computing averages and residuals

Suppose that we arrived at a solution to the discretized equilibrium equation (75),

82 $$\begin{aligned} \text {div}_N\,{{\,\mathrm{\mathcal {\varvec{S}}}\,}}(\cdot , \bar{\varvec{\varepsilon }} + \nabla _N \varvec{ u}) = 0, \end{aligned}$$

or, equivalently, to the Lippmann–Schwinger equation (81). The quantity of interest for computational homogenization techniques is to compute the apparent stress (18)

83 $$\begin{aligned} {{\,\mathrm{\mathcal {\varvec{S}}}\,}}^{\text {app}}(\bar{\varvec{\varepsilon }}) = \left\langle { {{\,\mathrm{\mathcal {\varvec{S}}}\,}}(\cdot , \bar{\varvec{\varepsilon }} + \nabla \varvec{ u})} \right\rangle _Y, \end{aligned}$$

or, to be more precise, an approximation thereof. The naive approach would be to consider the solution $\varvec{ u}\in \texttt {S}_N(Y;\mathbb {R}^3)$ to the discrete equilibrium problem (82), evaluate the stress

84 $$\begin{aligned} \varvec{\sigma }_N(\varvec{ x}_{\textbf{j}}) = {{\,\mathrm{\mathcal {\varvec{S}}}\,}}(\varvec{ x}_{\textbf{j}}, \bar{\varvec{\varepsilon }} + \nabla \varvec{ u}(\varvec{ x}_{\textbf{j}})), \quad \varvec{ x}_{\textbf{j}} \in Y_N, \end{aligned}$$

at the point of the discrete grid (40), and to compute the mean. However, this strategy is not recommended for two reasons, to be explained shortly. Rather, for an arbitrary element $\varvec{\tau }\in L^2(Y;\mathbb {R}^{3 \times 3})$, we define the consistent discrete average

85 $$\begin{aligned} \left\langle {\varvec{\tau }} \right\rangle _{Y_N} = \left. \sum _{\varvec{ x}_{\textbf{j}} \in Y_N} w_{\textbf{j}} \varvec{\tau }(\varvec{ x}_{\textbf{j}}) \Big / \sum _{\varvec{ x}_{\textbf{j}} \in Y_N} w_{\textbf{j}} \right. , \end{aligned}$$

which accounts for the weights (47) *and* makes sure that the mean preserves constants. As a direct consequence of the definition, we obtain the property

86 $$\begin{aligned} \left\langle {\bar{\varvec{\varepsilon }} + \nabla \varvec{ u}} \right\rangle _{Y_N} = \left\langle {\bar{\varvec{\varepsilon }}} \right\rangle _{Y_N} + \left\langle {\nabla \varvec{ u}} \right\rangle _{Y_N} = \bar{\varvec{\varepsilon }} \end{aligned}$$

for any $\bar{\varvec{\varepsilon }} \in \text {Sym}(3)$ and $\varvec{ u}\in \texttt {S}_N(Y;\mathbb {R}^3)$. Here, we used, on the one hand, the definition (85)

87 $$\begin{aligned} \left\langle {\bar{\varvec{\varepsilon }}} \right\rangle _{Y_N} = \left. \sum _{\varvec{ x}_{\textbf{j}} \in Y_N} w_{\textbf{j}} \bar{\varvec{\varepsilon }} \Big / \sum _{\varvec{ x}_{\textbf{j}} \in Y_N} w_{\textbf{j}} \right. = \bar{\varvec{\varepsilon }}, \end{aligned}$$

and, on the other hand, that the second term vanishes

88 $$\begin{aligned} \left\langle {\nabla \varvec{ u}} \right\rangle _{Y_N} = \left. \sum _{\varvec{ x}_{\textbf{j}} \in Y_N} w_{\textbf{j}} \nabla \varvec{ u}(\varvec{ x}_{\textbf{j}}) \Big / \sum _{\varvec{ x}_{\textbf{j}} \in Y_N} w_{\textbf{j}} \right. = 0, \end{aligned}$$

due to the exact-quadrature property (48).

The second advantage of the definition (85) is the validity of the Hill-Mandel formula

89 $$\begin{aligned} \left\langle { \langle \varvec{\sigma }_N, \bar{\varvec{ F}} + \nabla \varvec{ v}\rangle } \right\rangle _{Y_N} = {\left\langle {\left\langle { \varvec{\sigma }_N} \right\rangle _{Y_N} : \bar{\varvec{ F}}} \right\rangle } \end{aligned}$$

for the stress (84) associated to an equilibrium solution (82) and arbitrary $\bar{\varvec{ F}} \in \mathbb {R}^{3 \times 3}$ as well as $\varvec{ v}\in \texttt {S}_N(Y;\mathbb {R}^3)$. The identity (89) follows from

90 $$\begin{aligned} \left\langle { \langle \varvec{\sigma }_N, \bar{\varvec{ F}} + \nabla \varvec{ v}\rangle } \right\rangle _{Y_N} ={ \left\langle { \varvec{\sigma }_N, \bar{\varvec{ F}}} \right\rangle _{Y_N} }+ \left\langle { \langle \varvec{\sigma }_N, \nabla \varvec{ v}\rangle } \right\rangle _{Y_N} \end{aligned}$$

and the definition (61) of the discrete divergence which implies that the second term vanishes in view of the equilibrium condition (82).

We may also benefit from the identity (86) when assessing convergence of the basic scheme [65, 66]

91 $$\begin{aligned} \varvec{ F}^{k+1} = \bar{\varvec{\varepsilon }} - \Gamma ^0_N : \left[ {{\,\mathrm{\mathcal {\varvec{S}}}\,}}(\cdot , \varvec{ F}^k)-\mathbb {C}^0: \varvec{ F}^k\right] , \end{aligned}$$

associated to the Lippmann–Schwinger equation (81). Here, $\varvec{ F}^k$ denotes a deformation-gradient field which arises as the *k*-th iterate of the basic scheme. By the choice (86), the formula

92 $$\begin{aligned}&\left\langle {\Vert \varvec{ F}^{k+1} - \varvec{ F}^{k}\Vert ^2} \right\rangle _{Y_N}\!\nonumber \\&\quad =\left\langle {\Vert \varvec{ F}^{k+1}\Vert ^2} \right\rangle _{Y_N} - \left\langle {\Vert \varvec{ F}^{k}\Vert ^2} \right\rangle _{Y_N}\nonumber \\&\quad \!+ 2\left( \left\langle { \langle {{\,\mathrm{\mathcal {\varvec{S}}}\,}}(\cdot , \varvec{ F}^k), \varvec{ F}^k\rangle } \right\rangle _{Y_N} \right. \nonumber \\&\quad \left. \!- \left\langle \left\langle { {{\,\mathrm{\mathcal {\varvec{S}}}\,}}(\cdot , \varvec{ F}^k)} \right\rangle _{Y_N}, \left\langle {\varvec{ F}^k} \right\rangle _Y \right\rangle \right) \end{aligned}$$

holds [20, eq. (3.6)], which permits to exactly evaluate the residual of the basic scheme with only a single field in memory.

We wish to remark that with the consistent definition (86), the entire package of Lippmann–Schwinger technology [79], including mixed boundary conditions [40, 51] and advanced solvers like the conjugate-gradient method [7, 104] becomes available.

### Implementation

Our implementation makes use of the discrete cosine transform (DCT) and the discrete sine transform (DST) as implemented in the dedicated FFT library FFTW [25], i.e., we rely on the DCT-I in the form

93 $$\begin{aligned} X_j =&\, x_0 + (-1)^j x_{M-1} \nonumber \\&+ 2 \sum _{k=1}^{M-2} x_k \cos \left( \pi \, \frac{jk}{M-1}\right) ,\nonumber \\&j=0,1,\ldots ,M-1, \end{aligned}$$

as well as the DST-I

94 $$\begin{aligned} X_j =&\,2 \sum _{k=0}^{M-1} x_k \sin \left( \pi \, \frac{(j+1)(k+1)}{M+1}\right) , \nonumber \\&j=0,1,\ldots ,M-1, \end{aligned}$$

both for input signals

95 $$\begin{aligned} x = (x_0,x_1,\ldots ,x_{N-1}) \end{aligned}$$

of length *M*. The prefactors 1 and 2 in the DCT (93) and the DST (94) naturally ensure that boundary points are handled properly, i.e., incorporating the weights (42).

We apply the DCT and the DST to a one-dimensional input signal $x=(x_0,x_1,\ldots ,x_{N-1})$ of length *N* as follows:

1. We apply the DCT (93) to the entire input signal *x* for $M = N$ and call the operation DCT.
2. We apply the DST (94) to the shortened input signal $x'=(x_1,x_2,\ldots ,x_{N-2})$ for $M=N-2$, leaving the entries $x_0$ as well as $x_{N-1}$ untouched and call the operation DST.

A pseudo-code for the simplest basic scheme is given in Algorithm 1.
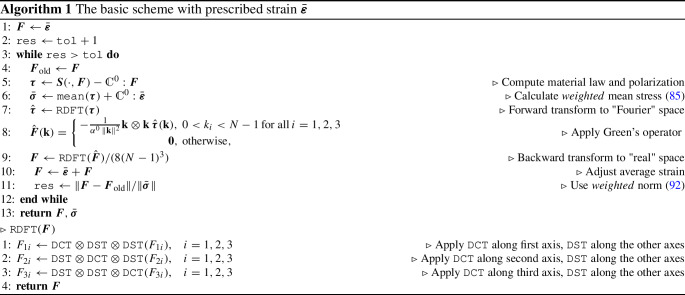


Compared to a standard basic scheme in FFT-based computational homogenization [65, 66], the following differences are apparent:

1. The implementation operates on the displacement gradient instead of the strain.
2. Discrete cosine and sine transforms are used instead of the FFT of real-valued data.
3. Weighted averages are considered.
4. Averages are not obtained via certain Fourier coefficients, and the mean strain is not imposed via manipulating a Fourier coefficient.
5. A whole number of frequencies are automatically set to zero (all those on the boundary of the box), reflecting the nature of the sine polynomial (39).

Apart from these changes, the implementation is straightforward. In particular, Dirichlet boundary conditions may be integrated into a working FFT-based computational homogenization code with relative ease. Also, most well-established extensions continue to work for this modification.

We conclude this section with a few remarks

1. For a linear elastic material ${{\,\mathrm{\mathcal {\varvec{S}}}\,}}\equiv \mathbb {C}$ satisfying the conditions
  96 $$\begin{aligned} \alpha _- \Vert \varvec{\varepsilon }\Vert ^2 \le \varvec{\varepsilon }: \mathbb {C}(\varvec{ x}) : \varvec{\varepsilon }\le \alpha _+ \Vert \varvec{\varepsilon }\Vert ^2 \end{aligned}$$
  for all
  $$\begin{aligned} \varvec{ x}\in Y \quad \text {and} \quad \varvec{\varepsilon }\in \mathbb {R}^{3 \times 3}_{\texttt {sym}} \end{aligned}$$
  with positive constants $\alpha _\pm$, the reference constant (27) should be chosen as follows:
  97 $$\begin{aligned} \alpha _0 = \frac{\alpha _+ + \alpha _-/2}{2}. \end{aligned}$$
  The factor 1/2 accompanying $\alpha _-$ is a consequence of Korn’s inequality [23]
  98 $$\begin{aligned}&\frac{1}{2} \int _Y \Vert \nabla \varvec{ u}\Vert ^2 \, d\,\varvec{ x}\nonumber \\&\quad \le \int _Y \Vert \nabla ^s \varvec{ u}\Vert ^2 \, d\,\varvec{ x}\nonumber \\&\quad \le \int _Y \Vert \nabla \varvec{ u}\Vert ^2 \, d\,\varvec{ x}, \end{aligned}$$
  valid for all $\varvec{ u}\in H^1_0(Y;\mathbb {R}^3)$. Similar considerations apply to more general non-linear constitutive laws [78]. The factor 1/2 in the estimate (98) implies that the condition number of the preconditioned linear system will be increased by a factor of two. In particular, we expect implications on the performance of the developed schemes, see Sec. 4.2 below.
2. It is possible to use implementations on the displacement in the sense of Kabel et al. [38], further refined in Grimm-Strele & Kabel [32]. In this way, the additional storage required for the displacement gradient is less severe. For the conjugate gradient method, $3 + 3 = 6$ displacement gradients need to be stored instead of $4 \cdot 3 = 12$, reducing the memory footprint by $50\%$.
3. Mixed boundary conditions [40, 51] are integrated in a straightforward way. Similarly, advanced solvers like fast [74, 77], conjugate-gradient methods [7, 104] or Newton-type methods [28, 96] are readily integrated.
4. Polarization methods in the sense of Monchiet-Bonnet [59, 60] may be explored, as well. However, there are two caveats. For a start, the simplified inversion formula in line 9 of Algorithm 1 no longer applies, and a refined formula needs to be used. Moreover, damping [80, 86] is required for the polarization schemes. Put differently, the Eyre–Milton scheme [21] does not converge, but the schemes by Michel–Moulinec–Suquet [56, 57] and Monchiet-Bonnet [59, 60] do.

-

## Computational investigations

### Setup

In this section, we investigate the numerical performance of applying Dirichlet boundary conditions using the approach harnessing the discrete sine and cosine transforms and compare the performance to the conventional approach with periodic boundary conditions. We implemented the Dirichlet boundary conditions into an in-house FFT-based homogenization code [79] written in Python with Cython extensions for performance-critical sections of code. The additional implementation effort consists of:

1. Replacing FFTs by appropriate DSTs/DCTs,
2. Adaptations to the preconditioner in the manner shown for the simple basic scheme in Algorithm 1,
3. Extraction/setting the mean of a field requires more work than accessing/manipulating a single Fourier frequency,
4. Accounting for appropriate weights (85) when computing mean values.

Consistent with the preexisting code base, we employ the FFTW [24] library to compute the (discrete) Fourier transforms, retaining comparability with the periodic case and enabling straightforward thread-parallel evaluation of the transforms.

Runtimes were recorded on a $2\times 48$-core AMD EPYC CPU with $1024\textrm{GB}$ of RAM. The computations were performed using the material parameters listed in Table 1

**Table 1:** Material parameters of materials used in the numerical studies and corresponding reference

Name	*E* in $\textrm{GPa}$	$\nu$	$k_1$ in $\textrm{GPa}$	$k_2$ in $\textrm{GPa}$	*m*	References
E-glass	72.0	0.22	–	–	–	[14]
UPPH resin	3.4	0.385	–	–	–	[43]
Polyamide	2.1	0.3	129	32.7	319.4	[14]

The E-glass and the UPPH resin are modeled as isotropic linear elastic, whereas the mechanical behavior of the polyamide matrix is governed by $J_2$-plasticity with isotropic exponential-linear hardening

99 $$\begin{aligned} \sigma _0(p)=\sigma _Y + k_1 p + k_2(1-\exp (-mp)). \end{aligned}$$

### Computational performance

**Fig. 2:**
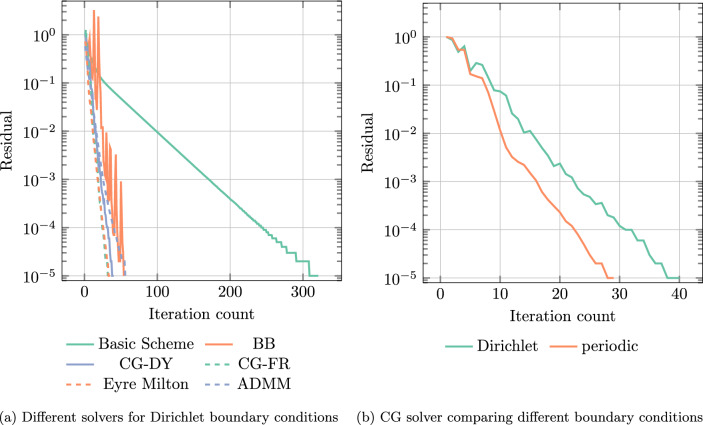
Residual versus iteration count for a single E-glass sphere in an UPPH matrix on a $256^3$ grid

Maybe the most striking advantage of the proposed novel treatment of Dirichlet boundary conditions in FFT-based computational micromechanics consists of retaining the full compatibility with the entire bouquet of previously established Lippmann–Schwinger technology. For instance, all the previously developed solvers become available.

To demonstrate this advantage, we consider a single E-glass sphere in an UPPH matrix, shown in Fig. 5d, and plot the residual versus iterations for different solvers in Fig. 2a for Dirichlet boundary conditions and a $256^3$ grid.

The basic scheme converges, and requires 321 iterations to achieve the prescribed tolerance of $10^{-5}$. With a total of 54 iterations, the Barzilai-Borwein (BB) [75] method requires only a fraction of the iterations of the basic scheme. However, we observe a non-monotonic decrease of the residual, a property which is well-known for the BB scheme [75]. Among the considered solution schemes, the lowest iteration count is achieved by the nonlinear conjugate gradient (CG) method where only 40 iterations are necessary to reach the desired tolerance with the added benefit of the steadily decreasing residual. Here, we show the plots for two different methods of selecting the momentum parameter in the nonlinear CG scheme, which are the Dai-Yuan (CG-DY) and Fletcher-Reeves (CG-FR) method [77]. For this linear problem setting with finite material contrast, both methods achieve convergence within the same number of iterations. Polarization methods [59, 64, 80] may also be used and show their well-known remarkable performance. Here, we show the Eyre–Milton method [21, 86] and the Michel–Moulinec–Suquet scheme [56, 57].

The trick for handling Dirichlet boundary conditions that was introduced in the work at hand comes at a price - using a vector Laplacian preconditioner on a small-strain problem increases the condition number of the system matrix by a factor of two. This fact is also reflected in the choice of the reference constant in the basic scheme compared to the periodic case, see Eq. 97. Therefore, we expect the iteration count to be higher for the Dirichlet case compared to periodic boundary conditions. To investigate the implications, we consider the same single spherical inclusion and consider both Dirichlet and periodic boundary conditions and the CG solver. In fact, the linear conjugate gradient method judiciously selects the involved algorithmic parameters in an optimal fashion and thus grants us an insight into whether our Korn estimate (98) is actually too pessimistic or not. We observe in Fig. 2b that the iteration count for Dirichlet boundary conditions requires 40 iterations compared to the 29 iterations needed in the periodic case to achieve the tolerance of $10^{-5}$. Thus, about $38\%$ more iterations are required for Dirichlet boundary conditions.

On the theoretical side, the classical worst-case bound for the iteration count of the linear CG for fixed tolerance involves the square root of the condition number of the (positive definite and symmetric) linear operator [68, eq. (5.35)]. Korn’s inequality (98) predicts that the condition number for our Laplace-preconditioned small-strain problem is at most twice as high as if we used a small-strain preconditioner (which appears to be unavailable by Fourier methods, however). As the considered bounds for Dirichlet and periodic boundary conditions actually only involve material parameters, they may be compared. Due to the additional factor two in the condition number estimate for Dirichlet boundary conditions compared to periodic boundary conditions, and the appearance of square root in the iteration-count estimate for CG, we expect an increase by a factor $\sqrt{2}\approx 1.44$ in the iteration count of CG when comparing Dirichlet and periodic boundary conditions. The observed $38\%$-increase is actually rather close to this theoretical prediction.

We take a closer look at the iteration counts and the runtimes for this setting, also in case of solvers with non-optimal parameter choices.

**Fig. 3:**
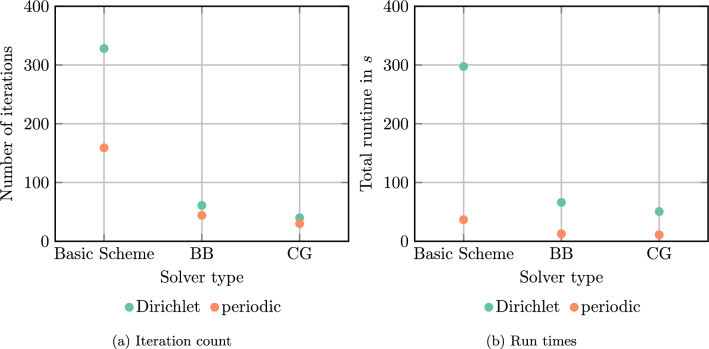
Iteration count and run times for a single E-glass sphere in an UPPH matrix on a $128^3$ grid and a prescribed tolerance of $10^{-5}$

For Dirichlet boundary conditions, the basic scheme needs 328 iterations, wheras only 159 iterations are required for periodic boundary conditions, see Fig. 3a. Thus, the iteration count roughly doubles, as expected from the Korn estimate (98). For the Barzilai-Borwein scheme, 61 iterations are needed for Dirichlet boundary conditions compared to 44 iterations for periodic boundary conditions. The increase by $39\%$ also appears plausible accounting for Korn’s inequality (98). Last but not least, the linear conjugate gradient method requires 40 iterations for Dirichlet boundary conditions compared to 30 iterations for periodic boundary conditions, implying a 1/3-increase in iteration count.

With these observations at hand, we take a look at the total runtimes shown in Fig. 3b. The linear CG method takes around $51\,\textrm{s}$ for the Dirichlet case compared to $11\,\textrm{s}$ for the periodic case. The absolute difference in runtime is even more prominent for the basic scheme, where the runtime of $37\,\textrm{s}$ with periodic boundary conditions is contrasted by the runtime of $297\,\textrm{s}$ for the Dirichlet case. There is a number of factors which are responsible for this increase. For a start, more iterations are required for the Dirichlet case. Secondly, larger arrays need to be traversed both for applying the constitutive law, see line 5 in Algorithm 1, and for taking care of the action of the Eshelby-Green operator, see line 8 in Algorithm 1. A third issue arises when computing the trigonometric transforms. Recall that periodic boundary conditions are naturally used when applying FFTs in all directions, leveraging the vectorization capabilities of the FFTW. In contrast, imposing Dirichlet boundary conditions requires applying discrete sine and cosine transforms in different directions of the deformation gradient/stress. In this context, implementations of the DST-I and DCT-I transforms operate on a mirrored signal [24, 33], leading to another source of slowdown. In addition, the DST is only applied to a subset of the considered field, which compromises proper memory alignment. A fourth source of slowdown arises from the need to compute averages over the field in the case of Dirichlet boundary conditions, rather than being able to read off the average from the zeroth frequency in the periodic case. Dually, imposing a macroscopic strain also requires more work in the Dirichlet case. Last but not least, the use of weighted quadrature slows down the implementation even more. This is particularly noticeable for the basic scheme, where we use the memory-efficient convergence criterion [20, eq. (3.6)] which requires computing a Hill-Type “work” term $\left\langle {\varvec{\sigma }:\varvec{ F}} \right\rangle _Y$ using the proper weights.

To sum up, we observe an increase in runtime, and the reasons for this increase are rather clear. We are confident that subsequent work may introduce ideas to reduce the computational overhead.

We close this section by resolution studies.

**Fig. 4:**
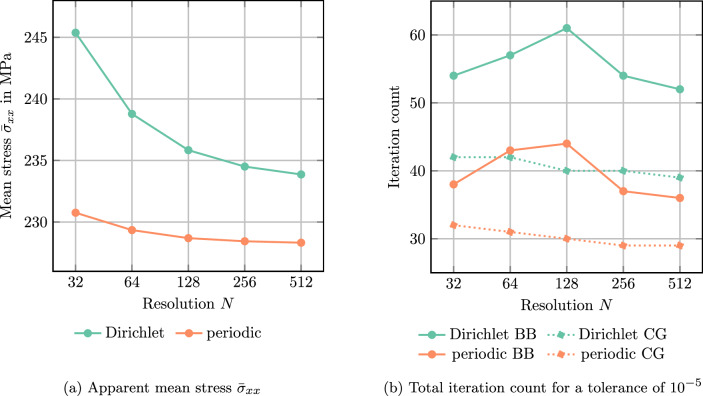
Resolution studies for the single spherical E-glass inclusion in an UPPH matrix under $5\%$ uniaxial extension in *x*-direction

We revisit the single spherical inclusion under uniaxial extension in *x*-direction with $5\%$ strain. The cubic domain is resolved by $N^3$ voxels for varying voxel count *N*. First, we monitor the mean normal stress in *x*-direction, see Fig. 4a. For both Dirichlet and periodic boundary conditions, the computed mean stress decreases with increasing voxel count. However, Dirichlet boundary conditions lead to consistently higher mean stresses than periodic boundary conditions. This situation is expected for the continuous setting, as variational arguments imply such an outcome [34, 36]. In fact, similar arguments could be applied to the Moulinec–Suquet discretization despite the quadrature-induced non-consistency.

Figure 4b shows the total number of iterations up to convergence with an error tolerance of $10^{-5}$ for two solvers, the Barzilai-Borwein (BB) method as well as the Conjugate Gradient (CG-DY) scheme. For the BB solver, we observe a fluctuation in the iteration count for both types of boundary conditions. As discussed previously, the iteration count for the Dirichlet case is consistently higher than for the periodic case. Nevertheless, the iteration count does not increase with increasing resolution. Despite the strongly fluctuating residual for the BB solver, see Fig. 2a, the highs and lows of the iteration count for varying resolution match for the two considered boundary conditions.

For the CG solver, we observe a slight yet steady decrease in iteration count for both types of boundary conditions. As for BB, imposing Dirichlet boundary conditions leads to consistently higher iteration counts.

To sum up, we confirm the theoretical expectations that the novel Lippmann–Schwinger solvers for Dirichlet boundary conditions in mechanics lead to an iteration count which is bounded independently of the resolution, but depends only on the material contrast.

### Influence of the boundary conditions

**Fig. 5:**
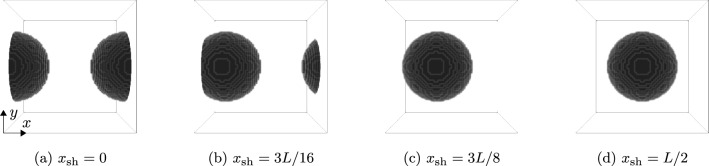
Spherical inclusion in a cubic cell $[0,L]^3$ with shift $x_\textrm{sh}$ in *x*-direction

To make the influence of the Dirichlet boundary conditions on the local fields more tangible, we consider the following simple numerical experiment involving a single spherical E-glass inclusion. For periodic boundary conditions, the effective properties are invariant towards shifting the position of the spherical inclusion, as long as that shift is an integer number of voxels. In particular, having the ball intersect the domain boundary does not influence the results. For the Dirichlet case, this invariance does not hold, as the microstructure is “clamped” on the domain boundaries. Accordingly, we compute the apparent properties and local fields using both periodic and Dirichlet boundary conditions on a cubic domain with edge length *L*, via the FFT and DCT/DST transforms, respectively. A single spherical inclusion of radius *L*/4 is shifted in *x*-direction by a certain amount from its initial position where the center of the sphere is at $x_\textrm{sh}=0$, i.e., centered on the domain boundary. Four spherical inclusions shifted by different amounts are shown in Fig. 5. Note that the microstructure itself is still periodic even in cases where the spherical inclusion overlays the domain boundary.

**Fig. 6:**
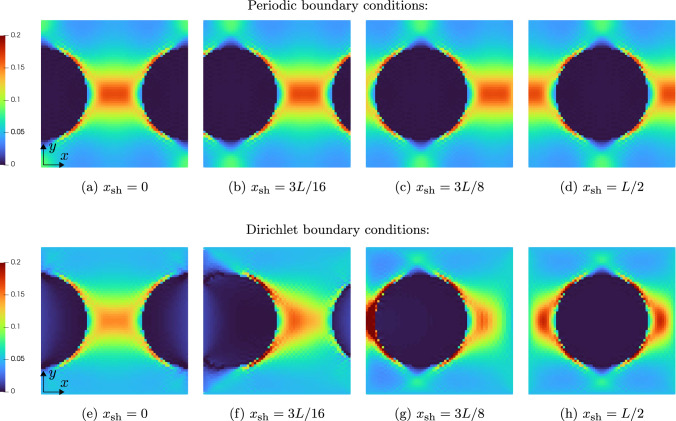
Slice of the local strain field $\varepsilon _{xx}$for the shifted spherical inclusion on a $128^3$ grid and $5\%$ uniaxial extension in *x*-direction

We consider the shifted spherical inclusions to be embedded a polyamide matrix governed by $J_2$-plasticity, see Table 1. The specimen is loaded by uniaxial extension up to a maximum of $5\%$ in *x*-direction, and we consider ten equidistant (monotonic) load step steps. Fig 6 shows a slice of the 3D local strain fields at the final load for different shifts of the sphere and the two considered boundary conditions for the displacement field.

We observe that for the periodic boundary conditions, the local strain fields do not change when shifting of the sphere, as expected. As a characteristic of the Moulinec–Suquet discretization, significant ringing artifacts occur, also in the periodic case, especially in the softer matrix material around the edges of the sphere. These artifacts, however, do not interfere with the accuracy of the discretization - both the local field and the effective stresses converge with the same rate [82] as when considering finite elements or a regular grid [102].

In contrast, imposing Dirichlet boundary conditions for the displacement fluctuation leads to significantly higher strains in case the spherical inclusion is cut by the domain boundary, see the bottom row in Fig. 6. Let us take a closer look at the different local fields.

In Fig. 6e, the sphere is clamped by the boundary. As the sphere is stiffer than the matrix material, the local solutions fields do not differ significantly compared to the respective strains for periodic boundary conditions in Fig. 6a. In fact, the maximum local strain in the middle of the domain is lower for the Dirichlet case than for the periodic case. Shifting the spherical inclusion to the right by 3*L*/16, see Fig. 6f, leads to completely different effects. In this case, the sphere is only partially clamped by the Dirichlet boundary condition and therefore a stronger increase in local strains is observable close to the sphere-matrix interface in the center of the domain. Additionally, a region of rather low local strains emerges in the matrix above and below the sphere on the left hand side, caused by the transverse contraction of the left half of the sphere as it is subjected to a loading in positive *x*-direction.

For the next scenario, the spherical inclusion is moved so far into the volume element that is does not intersect the boundary anymore, i.e., the sphere is not clamped directly, see Fig. 6g. We observe an increase of the local strains in the matrix material close to the boundary on the left hand side of the field. In contrast, on the right hand side of the local field, i.e., where no inclusion is present, the strains are noticeably lower than at the same location of the field with periodic boundary conditions shown in Fig. 6c.

By placing the sphere in the center of the domain, we see another change in the local strain field in Fig. 6d. In fact, the stress field is again perfectly symmetric, but differs noticeably from the field with periodic boundary conditions shown in Fig. 6d. The local strains at the boundary on the volume element are visibly decreased compared to the periodic case which is caused by the clamping of the displacement on the boundary in the Dirichlet case.

Concerning the aforementioned ringing artifacts, we notice a slight increase of these artifacts for the Dirichlet case, which are presumably caused by the increased levels of local strain. This amplification is especially noticeable when comparing Fig. 6c, g, where areas of high local strains lead to significantly more ringing in the Dirichlet case.

In addition to the local solution fields, we also investigate the mean stresses for the shifted-sphere microstructures, discretized by $128^3$ voxels.

**Fig. 7:**
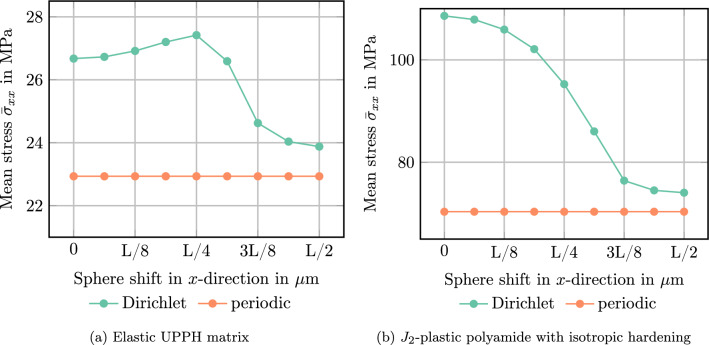
Mean stress under uniaxial loading for a single shifted E-glass-sphere

We consider two scenarios for the matrix material: an elastic UPPH matrix, shown in Fig. 7a, and an elastoplastic polyamide matrix, shown in Fig. 7b.

Not surprisingly, the invariance under shifting for the periodic boundary conditions that we observed for the local solution fields is also reflected in the computed mean stresses. As expected, the computed mean stresses do not change for all considered shifts.

In contrast, for the Dirichlet case and the elastic matrix material, see Fig. 7a, the mean stresses are significantly higher than in the periodic case in case the center of the spherical inclusion is not shifted, i.e., the sphere is located right on the domain boundary. Interestingly, shifting the sphere in *x*-direction leads to an increase in mean stresses. Only when the spherical inclusion itself is not clamped by the domain boundary anymore, i.e. starting from an *x*-shift of 5*L*/8 for a sphere of radius *L*/4, the mean stress gradually decreases. Nevertheless, the boundary condition influences the mean stresses even when the inclusion is perfectly centered in the domain for an *x*-shift of *L*/2 where the mean stress is still higher than the mean stress for the periodic case. Even though the sphere itself is not clamped anymore, the matrix material still is.

For the matrix material with isotropic hardening, we observe a different behavior in Fig. 7b. In contrast to the elastic case, no increase in mean stress is visible when moving the sphere in *x*-direction. The relative increase in mean stress between a sphere shift of 0 and a sphere shift *L*/2 is more significant for the matrix material with isotropic hardening. This is rooted in the matrix material’s ability to reduce local stress peaks by yielding. For the centered sphere in the case of the elastoplastic matrix model, the difference in mean stress between Dirichlet and periodic boundary conditions is less significant compared to the elastic case.

**Fig. 8:**
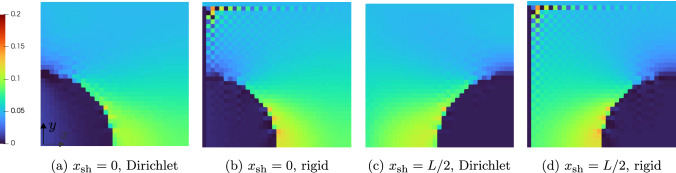
Upper left quadrant of a slice of the local strain field $\varepsilon _{xx}$for the shifted spherical inclusion and $5\%$ uniaxial extension in *x*-direction with Dirichlet boundary conditions as well as periodic boundary conditions in combination with a rigid outer layer

Another strategy to emulate Dirichlet boundary conditions is to add a rather stiff outer layer to the domain boundaries [3, 30]. Figure 8 shows a slice of the local stress fields for the solution fields of a shifted ball with Dirichlet boundary conditions (Fig. 8a, c) as well a solution field with periodic boundary conditions but a rigid outer layer with a rather high ($1\cdot 10^7\,\textrm{GPa}$) Young’s modulus (Fig. 8b, d). We observe that for the rigid outer layer, the general solution field on the interior of the domain is quite similar to the solution field with Dirichlet boundary conditions. Nevertheless, for the rigid outer layer strong ringing artifacts appear in the corner of the solution field caused by the significant material contrast. For the domain under consideration, our approach to imposing Dirichlet boundary conditions converges in 49 CG iterations, whereas the boundary-layer strategy requires 464 CG iterations to converge. This difference is rooted in the high material contrast for the almost rigid outer layer, leading to an increased condition number which is even more unfavorable than the increase inflicted by the DST/DST approach.

### Applications to materials with random microstructure

**Fig. 9:**
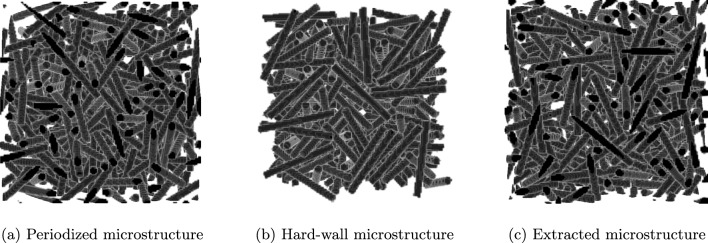
Examples for different types of fiber microstructures on a $128^3$ grid

When considering material with random microstructures, modern image-based characterization techniques like micro-computed tomography ($\mu$-CT) permit to gain in-depth knowledge of the underlying arrangement of the constituent phases on the microstructural scale. The obtained images may be complemented by a stochastic model of the microstructure to increase the fidelity in the subsequently computed effective material response. There are different ways to generate statistically similar microstructure, hopefully in such a way that the characteristic features of the real material are matched [71].

In the section at hand, we investigate the interplay of imposing Dirichlet boundary conditions and different ways to generate microstructures. To this end, we synthesized microstructure ensembles of three types. All three of them comprise E-glass fibers with an aspect ratio of ten embedded in an elastic UPPH matrix. The first type of microstructures ensemble we consider is periodic microstructures, i.e., those microstructures where the microstructure is perfectly periodic on the considered unit cell. A top view of an exemplary microstructure is shown in Fig. 9a.

As the second type of microstructure ensemble we consider microstructures with a hard-wall boundary condition, shown in Fig. 9b. For this microstructure type, the fibers are placed in such a way that no fiber is allowed to protrude the domain. Therefore, on the domain boundary, mostly matrix material is found. This emulates real samples molded to exactly the size of the sample, for instance.

The last microstructure type we investigate is the extracted type shown in Fig. 9c. In this case, the synthesized microstructure is extracted from a larger domain, emulating a (non-periodic) sample cut from a real microstructure.

**Fig. 10:**
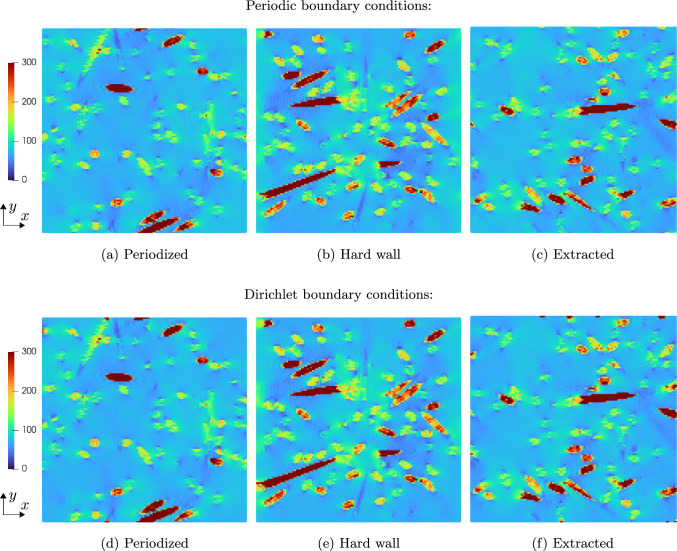
Local stress field $\sigma _{xx}$ in $\textrm{MPa}$ under uniaxial extension in *x*-direction—boundary condition versus microstructure type, see Fig. 9

We computed the apparent stiffnesses on a $128^3$ grid. To get an insight into the local stresss fields, we compare periodic and Dirichlet boundary conditions for the displacement-fluctuation field on a single realization of the three considered microstructure ensembles, see Fig. 10. For periodic boundary conditions and a periodic microstructure, shown in Fig. 10a, we observe no effects at the domain edges. However, the ringing artifacts representative for the Moulinec–Suquet discretization,i.e, the discretization via trigonometric polynomicals, become apparent.

In contrast, Dirichlet boundary conditions applied for the periodic microstructure, Fig. 10d, leads to increased local stresses in the fibers intersecting the domain boundary. These local stresses are significantly higher than for periodic boundary conditions.

For the microstructure with hard walls, where no fibers protrude into the domain boundary, we observe that the periodic and Dirichlet boundary conditions, see Fig. 10b, e, respectively, behave rather similarly. In fact, only slightly higher stresses emerge for Dirichlet boundary condition. Ringing is equally present for both types of boundary condition.

Last but not least, we consider the case of extracted ensembles. Figure 10c shows that periodic boundary conditions lead to lower local stresses on the domain boundary. In contrast, as shown in Fig. 10f, Dirichlet boundary conditions imply local stress peaks, especially at the top and on the right hand side of the domain where parts of the fiber barely protrude into the domain from above.

In addition to monitoring the local solution fields, we take a closer look at the apparent properties obtained for different types of microstructure ensemble in combination with the two different types of boundary conditions.

**Fig. 11:**
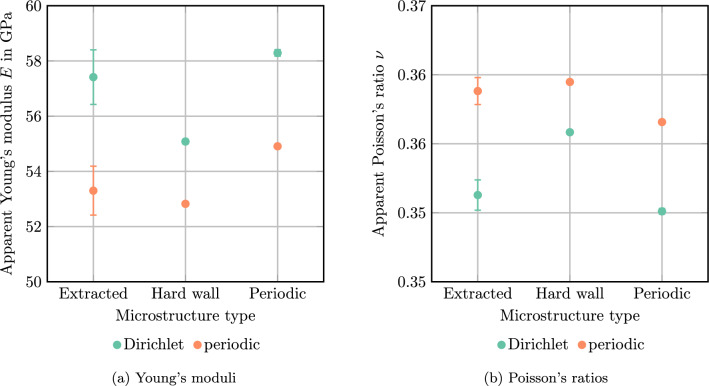
Apparent effective isotropic moduli of the volume element computed by isotropic approximation of the apparent stiffness for different types of microstructure ensembles

In total, we computed 20 samples for each combination of boundary condition and microstructure ensemble on a $128^3$ domain. For each sample, we computed the apparent stiffness matrix via six independent applied mean strains, i.e., three normal loadings in the respective coordinate directions as well as the three shear-load scenarios in all three coordinate plane. Subsequently, we extracted the apparent engineering constants such as the isotropic Young’s modulus and Poisson’s ratio.

In Fig. 11, the computed apparent Young’s modulus is shown for different types of microstructure ensembles for a glass fiber microstructure. We observe that the apparent stiffness and therefore the approximated Young’s modulus is higher for the Dirichlet boundary condition. This standard deviation, marked by the error bars in the plot is only noticeable for the extracted microstructure type. In that case, the volume fraction of the sample fluctuates more strongly, whereas the volume fraction is fixed for both the hard wall and the periodic microstructures.

We observe that the difference in apparent Young’s modulus is the largest for the extracted microstructure, where the value is higher for the Dirichlet boundary condition. For the hard-wall microstructure on the other hand, the difference is the smallest. This effect is a direct consequence of no fibers interpenetrating the domain boundary. Therefore, the Dirichlet boundary condition only influences the soft matrix material, reducing the impact of the boundary condition.

Finally, we see that the periodic microstructure yields the highest apparent Young’s modulus for both the Dirichlet and periodic boundary conditions. Like for the extracted microstructure, fibers can intersect the domain boundaries, therefore the Young’s modulus is influenced by the Dirichlet boundary condition. The same effect is visible for the approximated Poisson’s ratio in Fig. 11b, where the Dirichlet boundary conditions throughout yield lower apparent ratios than the periodic boundary condition. This is caused by the fixed boundaries in the Dirichlet case limiting the transverse contraction of the microstructure.

## Conclusions

In this work, we introduced a framework for applying Dirichlet boundary conditions for Lippmann–Schwinger solvers and computational micromechanics at small strains.

To this end, we started with the continuous setting, used a representation of the displacement-flucation field via a sine series and showed that apparent difficulties with the shear strain components can be circumvented by using a finite-strain Lippmann–Schwinger equation, i.e., employing Green’s function for the vector Laplacian instead of Green’s function for linear elasticity.

We worked out the details of this idea to obtain a computational scheme for the Moulinec–Suquet discretization, i.e., a discretization by trigonometric polynomials and where the trapezoid rule is used for quadrature. This top-down approach permitted us to naturally find the proper discrete sine and cosine transforms to use, and also made us aware of the proper weights to account for when computing averages.

The most striking advantage of the proposed piece of technology is its straightforward integrability into an existing FFT-based micromechanics solver ecosystem for inelastic and nonlinear materials. Essentially all existing features, including solvers, advanced macroscopic loadings [37, 40, 51] or composite voxels [31, 39, 41] can be used without much hassle.

The focus of the work at hand lay on the consistent derivation of the scheme from first principles, hopefully enabling researchers to build upon the introduced ideas in their own way and for their own applications. As a consequence of this focus, we did not put significant emphasis on optimizing the performance of the schemes. In fact, there is a certain overhead due to Korn’s inequality (98), and nine instead of six components of the fields need to be processed (e.g., for the transforms). Still, there is space for various optimizations, e.g., via dedicated displacement-based implementations [32, 52] or other tricks that we did not (yet) think of. In any case, we could show preliminary results which demonstrate the capabilities of the novel scheme.

The work at hand focused on Dirichlet boundary conditions for the Moulinec–Suquet discretization. However, enforcing Neumann boundary conditions, i.e., imposing normal stresses, via a similar approach seems plausible. Moreover, extending our ideas to different discretizations could be a promising direction of further study.

Last but not least, let us remark that our approach of enforcing boundary conditions is only viable on the exterior of a rectangular domain. Oftentimes this is the boundary of interest, but in some cases prescribing values on interior nodes is desirable, where Lagragian-type approaches like those introduced or Gélébart [30] or by To et al. [90] appear unavoidable.

## Data Availability

The data that support the findings of this study are available from the corresponding author upon reasonable request.

## Exact quadrature of real trigonometric polynomials

The purpose of this appendix is to shed some light on the quadrature rules (43) and (46) on the interval $[0,2\pi ]$. The transition to more general intervals [0, *L*] follows by a change of variables.

As usual, working with complex numbers helps to understand the matters more clearly. We consider trigonometric polynomials of order $M = 2P$

100 $$\begin{aligned} T(x) = \sum _{\xi =-P+1}^P \hat{T}(\xi ) \exp (2 \pi i \,x \xi ), \quad x \in [0,2\pi ], \end{aligned}$$

with a non-negative integer $P \in \mathbb {N}$. For trigonometric polynomials $T_1$ and $T_2$, Parseval’s Theorem [82, eq. (2.13)] holds in the form

101 $$\begin{aligned} \frac{1}{2\pi }&\int _0^{2\pi } T_1(x) \overline{T_2(x)} \, dx \nonumber \\&= \frac{1}{M} \sum _{k=0}^{M-1} T_1 \left( 2\pi \,\frac{k}{M} \right) \overline{T_2 \left( 2\pi \, \frac{k}{M} \right) } \end{aligned}$$

of an exact quadrature rule and where the bar indicates complex conjugation.

A sine polynomial

102 $$\begin{aligned} \phi (x) = \sum _{k=1}^{N-2} \hat{\phi }_k \sin \left( \pi \, kx\right) \end{aligned}$$

is a trigonometric polynomials of order $M = 2N-2$, i.e., with $P=N-1$. As the sine is an odd function, and the products of odd functions is even, we obtain, on the one hand

103 $$\begin{aligned} \frac{1}{2\pi } \int _0^{2\pi } \phi _1(x) \phi _2(x) \, dx = \frac{1}{\pi } \int _0^{\pi } \phi _1(x) \phi _2(x) \, dx \end{aligned}$$

for sine polynomials $\phi _1$ and $\phi _2$ of the form (102), where we also used that the sine function is real-valued. Inspecting the right-hand side of Eq. (101), we notice

104 $$\begin{aligned} \frac{1}{M}&\sum _{k=0}^{M-1} T_1 \left( 2\pi \,\frac{k}{M} \right) \overline{T_2 \left( 2\pi \, \frac{k}{M} \right) }\nonumber \\&= \frac{1}{N - 1} \sum _{k=1}^{N-2} \phi _1 \left( \frac{k\, \pi }{N-1} \right) \phi _2 \left( \frac{k\, \pi }{N-1} \right) , \end{aligned}$$

where we used, again, that the product of odd functions is even, and that the sine vanishes for $k=0$ and $k=N-1$. Thus, we arrived at the statement

105 $$\begin{aligned} \frac{1}{\pi }&\int _0^{\pi } \phi _1(x) \phi _2(x) \, dx \nonumber \\&= \frac{1}{N - 1} \sum _{k=1}^{N-2} \phi _1 \left( \frac{k\, \pi }{N-1} \right) \phi _2 \left( \frac{k\, \pi }{N-1} \right) , \end{aligned}$$

which we wanted to establish (43) for the sine case.

In addition to the sine case (102), we also investigate cosine polynomials

106 $$\begin{aligned} \psi (x) = \sum _{k=0}^{N-2} \hat{\psi }_k \cos \left( \pi \, kx\right) . \end{aligned}$$

These functions comprise trigonometric polynomials of order $M = 2N-2$, as well. For two cosine polynomials $\psi _1$ and $\psi _2$ of the form (106), being real and even leads to the identity

107 $$\begin{aligned} \frac{1}{2\pi } \int _0^{2\pi } \psi _1(x) \psi _2(x) \, dx = \frac{1}{\pi } \int _0^{\pi } \psi _1(x) \psi _2(x) \, dx. \end{aligned}$$

Taking a closer look at the right-hand side of equation (101), we observe

108 $$\begin{aligned}&\frac{1}{M} \sum _{k=0}^{M-1} T_1 \left( 2\pi \,\frac{k}{M} \right) \overline{T_2 \left( 2\pi \, \frac{k}{M} \right) }.\nonumber \\&\quad = \frac{1}{2(N-1)} \sum _{k=0}^{2N-3} \psi _1 \left( \pi \,\frac{k}{N-1} \right) \psi _2 \left( \pi \, \frac{k}{N-1} \right) .\nonumber \\&\quad = \frac{1}{2(N-1)} \left[ \psi _1(0)\psi _2(0) + 2\sum _{k=1}^{N-2} \psi _1 \left( \pi \,\frac{k}{N-1} \right) \right. \nonumber \\&\qquad \left. \cdot \,\psi _2 \left( \pi \, \frac{k}{N-1} \right) + \psi _1(\pi )\psi _2(\pi )\right] .\nonumber \\&\quad = \frac{1}{N-1} \left[ \frac{\psi _1(0)\psi _2(0)}{2} + \sum _{k=1}^{N-2} \psi _1 \left( \pi \,\frac{k}{N-1} \right) \right. \nonumber \\&\qquad \left. \cdot \,\psi _2 \left( \pi \, \frac{k}{N-1} \right) + \frac{\psi _1(\pi )\psi _2(\pi )}{2}\right] . \end{aligned}$$

Thus, we are led to the identity

109 $$\begin{aligned} \frac{1}{\pi } \int _0^{\pi }&\psi _1(x) \psi _2(x) \, dx = \frac{1}{N-1} \left[ \frac{\psi _1(0)\psi _2(0)}{2} \right. \nonumber \\&\quad + \sum _{k=1}^{N-2} \psi _1 \left( \pi \,\frac{k}{N-1} \right) \psi _2 \left( \pi \, \frac{k}{N-1} \right) \nonumber \\&\quad \left. + \frac{\psi _1(\pi )\psi _2(\pi )}{2}\right] , \end{aligned}$$

which implies the desired equation (43) directly for the cosine case ($k=0$ excluded)

110 $$\begin{aligned} \psi (x) = \sum _{k=1}^{N-2} \hat{\psi }_k \cos \left( \pi \, kx\right) \end{aligned}$$

and the result (via (46))

111 $$\begin{aligned}&\frac{\psi _1(0)\psi _2(0)}{2} + \sum _{k=1}^{N-2} \psi _1 \left( \pi \,\frac{k}{N-1} \right) \psi _2 \left( \pi \, \frac{k}{N-1} \right) \nonumber \\&\quad + \frac{\psi _1(\pi )\psi _2(\pi )}{2} = 0 \end{aligned}$$

by setting $\psi _1 \equiv \psi$ for the setup (110) and $\psi _2 \equiv 1$ in Eq. (109).

## Footnotes

1 The extension to general rectangular boxes $Y = [0,L_1] \times [0,L_2] \times [0,L_3]$ is straightforward, but comes at the expense of more involved notation.

## References

[1] Bargmann S, Klusemann B, Markmann J et al (2018) Generation of 3D representative volume elements for heterogeneous materials: a review. Prog Mater Sci 96:322–384

[2] Barzilai J, Borwein JM (1988) Two-point step size gradient methods. IMA J Numer Anal 8:141–148

[3] Bödeker F, Herr P, Biel A et al (2022) An FFT-based homogenization scheme for cohesive zones with an application to adhesives and the core material of thin metal sandwich plates. Theoret Appl Fract Mech 129:104186

[4] Bödeker F, Herr P, Moshfegh R et al (2022) A novel FFT-based homogenization scheme for cohesive zones. Procedia Struct Integrity 42:490–497

[5] Bhattacharya K, Suquet P (2005) A model problem concerning recoverable strains of shape-memory polycrystals. Proc R Soc A 461:2797–2816

[6] Bonnet G (2007) Effective properties of elastic periodic composite media with fibers. J Mech Phys Solids 55:881–899

[7] Brisard S, Dormieux L (2010) FFT-based methods for the mechanics of composites: a general variational framework. Comput Mater Sci 49(3):663–671

[8] Brisard S, Dormieux L (2012) Combining Galerkin approximation techniques with the principle of Hashin and Shtrikman to derive a new FFT-based numerical method for the homogenization of composites. Comput Methods Appl Mech Eng 217–220:197–212

[9] Chen Y, Gélébart L, Chateau C et al (2019) Analysis of the damage initiation in a SiC/SiC composite tube from a direct comparison between large-scale numerical simulation and synchrotron X-ray micro-computed tomography. Int J Solids Struct 161:111–126

[10] Chen Y, Vasiukov D, Gélébart L et al (2019) Fast Fourier transform solver for damage modeling of composite materials. JMST Adv 1:49–55

[11] Dame Carroll JR, Chandra A, Jones AS et al (2006) Airway dimensions measured from micro-computed tomography and high-resolution computed tomography. Eur Respir J 28(4):712–720 16870669 10.1183/09031936.06.00012405

[12] de Geus TW, Vondřejc J, Zeman J et al (2017) Finite strain FFT-based non-linear solvers made simple. Comput Methods Appl Mech Eng 318:412–430

[13] Despande VS, Fleck NA, Ashby MF (2001) Effective properties of the octet-truss lattice material. J Mech Phys Solids 49(8):1747–1769

[14] Doghri I, Brassart L, Adam L et al (2011) A second-moment incremental formulation for the mean-field homogenization of elasto-plastic composites. Int J Plast 27:352–371

[15] Dorn C, Schneider M (2019) Lippmann-Schwinger solvers for the explicit jump discretization for thermal computational homogenization problems. Int J Numer Methods Eng 118(11):631–653

[16] Drugan WJ, Willis JR (1996) A micromechanics-based nonlocal constitutive equation and estimates of representative volume element size for elastic composites. J Mech Phys Solids 44(4):487–524

[17] Eisenlohr P, Diehl M, Lebensohn RA et al (2013) A spectral method solution to crystal elasto-viscoplasticity at finite strains. Int J Plast 46:37–53

[18] Elliott JC, Dover SD (1982) X-ray microtomography. J Microscopy 126(2):211–213 10.1111/j.1365-2818.1982.tb00376.x 7086891

[19] Ernesti F, Schneider M (2021) A fast Fourier transform based method for computing the effective crack energy of a heterogeneous material on a combinatorially consistent grid. Int J Numer Methods Eng 122(21):6283–6307

[20] Ernesti F, Schneider M, Böhlke T (2020) Fast implicit solvers for phase field fracture problems on heterogeneous microstructures. Comput Methods Appl Mech Eng 363:112793

[21] Eyre DJ, Milton GW (1999) A fast numerical scheme for computing the response of composites using grid refinement. Eur Phys J Appl Phys 6(1):41–47

[22] Feng H, Zhao S (2020) FFT-based high order central difference schemes for three-dimensional Poisson’s equation with various types of boundary conditions. J Comput Phys 410:109391

[23] Friedrichs KO (1947) On the boundary value problems of the theory of elasticity and Korn’s inequality. Ann Math 48:441–471

[24] Frigo M, Johnson SG (1998) FFTW: An adaptive software architecture for the FFT. In: Proceedings 1998 IEEE international conference on acoustics speech and signal processing, vol 3. IEEE, pp 1381–1384

[25] Frigo M, Johnson SG (2005) The Design and Implementation of FFTW3. In: Proceedings of the IEEE, pp 216–231

[26] Fuka V (2015) PoisFFT—a free parallel fast Poisson solver. Appl Math Comput 267:356–364

[27] Gelebart L (2024) FFT-based simulations of heterogeneous conducting materials with combined non-uniform Neumann, periodic and Dirichlet boundary conditions. Eur J Mech A/Solids 105248

[28] Gélébart L, Mondon-Cancel R (2013) Non-linear extension of FFT-based methods accelerated by conjugate gradients to evaluate the mechanical behavior of composite materials. Comput Mater Sci 77:430–439

[29] Göküzüm FS, Nguyen LTK, Keip MA (2019) A multiscale FE-FFT framework for electro-active materials at finite strains. Comput Mech 64:63–84

[30] Gélébart L (2020) A modified FFT-based solver for the mechanical simulation of heterogeneous materials with Dirichlet boundary conditions. Comptes Rendus Mécanique 348(8–9):693–704

[31] Gélébart L, Ouaki F (2015) Filtering material properties to improve FFT-based methods for numerical homogenization. J Comput Phys 294:90–95

[32] Grimm-Strele H, Kabel M (2019) Runtime optimization of a memory efficient CG solver for FFT-based homogenization: implementation details and scaling results for linear elasticity. Comput Mech 64(5):1339–1345

[33] Grimm-Strele H, Kabel M (2021) FFT-based homogenization with mixed uniform boundary conditions. Int J Numer Methods Eng 122:7241–7265

[34] Hazanov S, Amieur M (1995) On overall properties of elastic heterogeneous bodies smaller than the representative volume. Int J Eng Sci 33(9):1289–1301

[35] Hill R (1963) Elastic properties of reinforced solids: some theoretical principles. J Mech Phys Solids 11(5):357–372

[36] Huet C (1990) Application of variational concepts to size effects in elastic heterogeneous bodies. J Mech Phys Solids 38(6):813–841

[37] Kabel M (2022) Mixed strain/stress gradient loadings for FFT-based computational homogenization methods. Comput Mech 70:281–308

[38] Kabel M, Böhlke T, Schneider M (2014) Efficient fixed point and Newton–Krylov solvers for FFT-based homogenization of elasticity at large deformations. Comput Mech 54(6):1497–1514

[39] Kabel M, Merkert D, Schneider M (2015) Use of composite voxels in FFT-based homogenization. Comput Methods Appl Mech Eng 294:168–188

[40] Kabel M, Fliegener S, Schneider M (2016) Mixed boundary conditions for FFT-based homogenization at finite strains. Comput Mech 57(2):193–210

[41] Kabel M, Fink A, Schneider M (2017) The composite voxel technique for inelastic problems. Comput Methods Appl Mech Eng 322:396–418

[42] Kanit T, Forest S, Galliet I et al (2003) Determination of the size of the representative volume element for random composites: statistical and numerical approach. J Mech Phys Solids 40(13–14):3647–3679

[43] Kehrer L, Wicht D, Wood JT et al (2018) Dynamic mechanical analysis of pure and fiber-reinforced thermoset- and thermoplastic-based polymers and free volume-based viscoelastic modeling. GAMM-Mitteilungen 41(1):1–16

[44] Kröner E (1977) Bounds for effective elastic moduli of disordered materials. J Mech Phys Solids 25(2):137–155

[45] Ladecký M, Leute RJ, Falsafi A et al (2023) An optimal preconditioned FFT-accelerated finite element solver for homogenization. Appl Math Comput 446:127835

[46] Lahellec N, Michel JC, Moulinec H et al (2003) Analysis of inhomogeneous materials at large strains using fast Fourier transforms. In: Miehe C (ed) IUTAM symposium on computational mechanics of solid materials at large strains, solid mechanics and its applications, vol 108. Springer, Netherlands, pp 247–258

[47] Lebensohn R, Rollett AD, Suquet P (2011) Fast Fourier transform-based modeling for the determination of micromechanical fields in polycrystals. JOM 63:13–18

[48] Lebensohn RA, Rollett AD (2020) Spectral methods for full-field micromechanical modelling of polycrystalline material. Comput Mater Sci 173:109336

[49] Leuschner M, Fritzen F (2018) Fourier-Accelerated Nodal Solvers (FANS) for homogenization problems. Comput Mech 62:359–392

[50] Li J, Tian XX, Abdelmoula R (2012) A damage model for crack prediction in brittle and quasi-brittle materials solved by the FFT method. Int J Fract 173:135–146

[51] Lucarini S, Segurado J (2019) An algorithm for stress and mixed control in Galerkin-based FFT homogenization. Int J Numer Methods Eng 119:797–805

[52] Lucarini S, Segurado J (2019) DBFFT: a displacement based FFT approach for non-linear homogenization of the mechanical behavior. Int J Eng Sci 114:103131

[53] Lucarini S, Cobian L, Voitus A et al (2022) Adaptation and validation of FFT methods for homogenization of lattice based materials. Comput Methods Appl Mech Eng 388:114223

[54] Ma R, Truster TJ (2019) FFT-based homogenization of hypoelastic plasticity at finite strains. Comput Methods Appl Mech Eng 349:499–521

[55] Matouš K, Geers MGD, Kouznetsova VG et al (2017) A review of predictive nonlinear theories for multiscale modeling of heterogeneous materials. J Comput Phys 330:192–220

[56] Michel JC, Moulinec H, Suquet P (2000) A computational method based on augmented Lagrangians and fast Fourier transforms for composites with high contrast. Comput Model Eng Sci 1(2):79–88

[57] Michel JC, Moulinec H, Suquet P (2001) A computational scheme for linear and non-linear composites with arbitrary phase contrast. Int J Numer Methods Eng 52:139–160

[58] Monchiet V (2015) Combining FFT methods and standard variational principles to compute bounds and estimates for the properties of elastic composites. Comput Methods Appl Mech Eng 283:454–473

[59] Monchiet V, Bonnet G (2012) A polarization-based FFT iterative scheme for computing the effective properties of elastic composites with arbitrary contrast. Int J Numer Methods Eng 89:1419–1436

[60] Monchiet V, Bonnet G (2013) Numerical homogenization of nonlinear composites with a polarization-based FFT iterative scheme. Comput Mater Sci 79:276–283

[61] Monchiet V, Bonnet G (2024) FFT based iterative schemes for composite conductors with uniform boundary conditions. Eur J Mech A Solids 103:105146

[62] Moos C (2013) An algorithm for damage mechanics based on the fast Fourier transform. Doctoral thesis (Dr.-Ing), Ruhr-Universität Bochum

[63] Morin L, Paux J (2024) A fast numerical method for the conductivity of heterogeneous media with Dirichlet boundary conditions based on discrete sine-cosine transforms. Comput Methods Appl Mech Eng 421:116772

[64] Moulinec H, Silva F (2014) Comparison of three accelerated FFT-based schemes for computing the mechanical response of composite materials. Int J Numer Methods Eng 97:960–985

[65] Moulinec H, Suquet P (1994) A fast numerical method for computing the linear and nonlinear mechanical properties of composites. Comptes Rendus de l’Académie des Sciences Série II 318(11):1417–1423

[66] Moulinec H, Suquet P (1998) A numerical method for computing the overall response of nonlinear composites with complex microstructure. Comput Methods Appl Mech Eng 157:69–94

[67] Mura T (1987) Micromechanics of defects in solids. Martinus Nijhoff, Dordrecht

[68] Nocedal J, Wright SJ (1999) Numerical optimization. Springer, New York

[69] Pahr DH, Zysset PK (2021) Influence of boundary conditions on computed apparent elastic properties of cancellous bone. Biomech Model Mechanobiol 7(6):463–476 10.1007/s10237-007-0109-7 17972122

[70] Risthaus L, Schneider M (2023) Imposing different boundary conditions for thermal computational homogenization problems with FFT- and tensor-train-based Green’s operator methods. Int J Numer Methods Eng e7423

[71] Sab K, Nedjar B (2005) Periodization of random media and representative volume element size for linear composites. Comptes Rendus Mécanique 333(2):187–195

[72] Sancho R, de Pedraza VR, Lafourcade P et al (2023) An implicit FFT-based method for wave propagation in elastic heterogeneous media. Comput Methods Appl Mech Eng 404:115772

[73] Schneider M (2015) Convergence of FFT-based homogenization for strongly heterogeneous media. Math Methods Appl Sci 38(13):2761–2778

[74] Schneider M (2017) An FFT-based fast gradient method for elastic and inelastic unit cell homogenization problems. Comput Methods Appl Mech Eng 315:846–866

[75] Schneider M (2019) On the Barzilai-Borwein basic scheme in FFT-based computational homogenization. Int J Numer Methods Eng 118(8):482–494

[76] Schneider M (2019) On the mathematical foundations of the self-consistent clustering analysis for non-linear materials at small strains. Comput Methods Appl Mech Eng 354:783–801

[77] Schneider M (2020) A dynamical view of nonlinear conjugate gradient methods with applications to FFT-based computational micromechanics. Comput Mech 66:239–257

[78] Schneider M (2020) Lippmann–Schwinger solvers for the computational homogenization of materials with pores. Int J Numer Methods Eng 121(22):5017–5041

[79] Schneider M (2021) A review of non-linear FFT-based computational homogenization methods. Acta Mech 232:2051–2100

[80] Schneider M (2021) On non-stationary polarization methods in FFT-based computational micromechanics. Int J Numer Methods Eng 122(22):6800–6821

[81] Schneider M (2022) Superaccurate effective elastic moduli via postprocessing in computational homogenization. Int J Numer Methods Eng 123(17):4119–4135

[82] Schneider M (2023) On the effectiveness of the Moulinec-Suquet discretization for composite materials. Int J Numer Methods Eng 124(14):3191–3218

[83] Schneider M, Wicht D (2023) Superconvergence of the effective Cauchy stress in computational homogenization of inelastic materials. Int J Numer Methods Eng 124(4):959–978

[84] Schneider M, Ospald F, Kabel M (2016) Computational homogenization of elasticity on a staggered grid. Int J Numer Methods Eng 105(9):693–720

[85] Schneider M, Merkert D, Kabel M (2017) FFT-based homogenization for microstructures discretized by linear hexahedral elements. Int J Numer Methods Eng 109:1461–1489

[86] Schneider M, Wicht D, Böhlke T (2019) On polarization-based schemes for the FFT-based computational homogenization of inelastic materials. Comput Mech 64(4):1073–1095

[87] Schneider M, Josien M, Otto F (2022) Representative volume elements for matrix-inclusion composites—a computational study on the effects of an improper treatment of particles intersecting the boundary and the benefits of periodizing the ensemble. J Mech Phys Solids 158:104652

[88] Segurado J, Lebensohn RA, LLorca J (2018) Chapter One—Computational homogenization of polycrystals. Adv Appl Mech 51:1–114

[89] Terada K, Hori M, Kyoya T et al (2000) Simulation of the multi-scale convergence in computational homogenization approaches. Int J Solids Struct 37(16):2285–2311

[90] To QD, Bonnet G, Nguyen-Thoi T (2021) Fourier transform approach to nonperiodic boundary value problems in porous conductive media. Int J Numer Methods Eng 122(18):4864–4885

[91] Vidyasagar A, Tutcuoglu AD, Kochmann DM (2018) Deformation patterning in finite-strain crystal plasticity by spectral homogenization with application to magnesium. Comput Methods Appl Mech Eng 335:584–609

[92] Vinogradov V, Milton GW (2008) An accelerated FFT algorithm for thermoelastic and non-linear composites. Int J Numer Methods Eng 76:1678–1695

[93] Vondřejc J (2014) Improved guaranteed computable bounds on homogenized properties of periodic media by Fourier–Galerkin method with exact integration. Int J Numer Methods Eng 107:1106–1135

[94] Vondřejc J, Zeman J, Marek I (2014) An FFT-based Galerkin method for homogenization of periodic media. Comput Math Appl 68(3):156–173

[95] Wathen A (2015) Preconditioning. Acta Numer 24:329–376

[96] Wicht D, Schneider M, Böhlke T (2020) On Quasi-Newton methods in FFT-based micromechanics. Int J Numer Methods Eng 121(8):1665–1694

[97] Wicht D, Schneider M, Böhlke T (2021) Anderson-accelerated polarization schemes for FFT-based computational homogenization. Int J Numer Methods Eng 122(9):2287–2311

[98] Wiegmann A (1999) Fast Poisson, fast Helmholtz and fast linear elastostatic solvers on rectangular parallelepipeds. Technical Report Lawrence Berkeley National Laboratory LBNL-43565:1–21

[99] Willot F (2015) Fourier-based schemes for computing the mechanical response of composites with accurate local fields. Comptes Rendus Mécanique 343:232–245

[100] Willot F (2020) The effective conductivity of strongly nonlinear media: the dilute limit. Int J Solids Struct 184:287–295

[101] Willot F, Abdallah B, Pellegrini YP (2014) Fourier-based schemes with modified Green operator for computing the electrical response of heterogeneous media with accurate local fields. Int J Numer Methods Eng 98:518–533

[102] Ye C, Chung ET (2023) Convergence of trigonometric and finite-difference discretization schemes for FFT-based computational micromechanics. BIT Numer Math 63:11

[103] Zeller R, Dederichs PH (1973) Elastic constants of polycrystals. Physica Status Solidi 55(2):831–842

[104] Zeman J, Vondřejc J, Novák J et al (2010) Accelerating a FFT-based solver for numerical homogenization of periodic media by conjugate gradients. J Comput Phys 229(21):8065–8071

[105] Zeman J, de Geus TWJ, Vondřejc J et al (2017) A finite element perspective on nonlinear FFT-based micromechanical simulations. Int J Numer Methods Eng 111:903–926

